# Structural Dynamics of Peptiplexes Formed between Cationic Cell-Penetrating Peptides and DNA: A Comparative Study on TAT-HIV and NLS-SV40T

**DOI:** 10.1021/acsabm.5c01567

**Published:** 2026-01-20

**Authors:** Lucas R. de Mello, Ibrahim A. Siddiq, Bianca B. M. Garcia, Ian W. Hamley, Karin A. Riske, Sang W. Han, Guillaume Tresset, Yves Lansac, Yun Hee Jang, Emerson R. da Silva

**Affiliations:** † Departamento de Biofísica, 28105 Universidade Federal de São Paulo, São Paulo 04062-000, Brazil; ‡ Department of Chemistry, 6816 University of Reading, Reading RG6 6AD, United Kingdom; § Department of Energy Science and Engineering, DGIST, Daegu 42988, Korea; ∥ Department of Experimental Research, Hospital Israelita Albert Einstein, São Paulo, São Paulo 05653-000, Brazil; ⊥ Université Paris-Saclay, CNRS, Laboratoire de Physique des Solides, 91405 Orsay, France; # GREMAN, UMR 7347, Université de Tours, CNRS, 37200 Tours, France

**Keywords:** cell-penetrating peptides (CPPs), nonviral gene delivery, peptide-DNA self-assembly, structural dynamics of soft biomaterials, supramolecular nucleic acid carriers, peptiplex-mediated DNA delivery

## Abstract

Biomembranes evolved to protect cells and regulate exchange, forming a powerful barrier to large, charged macromolecules such as nucleic acids. In recent years, this paradigm has been competently overturned by soft biomaterials based on cell-penetrating peptides (CPPs). Herein, we investigate and compare the structural dynamics of peptiplexes formed between DNA and two cationic CPPs, TAT-HIV and NLS-SV40T. Combining experimental approaches and molecular dynamics (MD) simulations, we examined peptiplexes across mesoscopic scales to elucidate their supramolecular assembly and correlate these features with cellular uptake. We found that peptiplexes based on TAT-HIV exhibit greater structural flexibility, adopting ordered secondary structures and self-assembling into clusters and nanofibrils. In contrast, NLS-SV40T/DNA complexes retain random coil configurations, forming globule-studded coiled nanoassemblies with internal 2D hexagonal columnar phases. Calorimetry data indicated that TAT-HIV/DNA complexation is more favorable and exothermic, whereas NLS-SV40T binding to DNA is weaker and endothermic. MD simulations supported the experiments by showing that NLS-SV40T moves across DNA strands, settling into major grooves, whereas TAT-HIV bridges major and minor grooves via persistent arginine-mediated H-bonds and stronger energetics. Cell uptake assays showed that NLS-SV40T/DNA peptiplexes are internalized comparatively more efficiently, likely due to their more compact organization and lower lytic potential. Conversely, TAT-HIV induces membrane damage, as observed by atomic force microscopy, suggesting that its stronger electrostatics and enhanced H-bonding capacity may contribute to lytic activity. The findings presented here bring mechanistic insights into the structural landscape of peptiplexes, improving the rationale that supports the design of peptide-mediated gene delivery materials.

## Graphical abstract

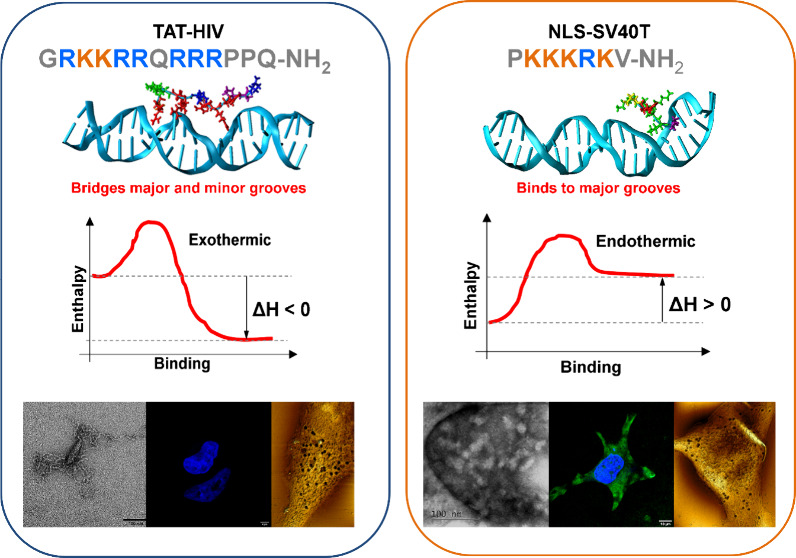

## Introduction

Cell membranes have evolved for nearly four billion years to define cell boundaries, protect intracellular compartments, and regulate exchange of matter and energy.^1^ The consequence of this evolutionary refinement is a highly dynamic barrier that poses a formidable challenge to the uptake of macromolecules, particularly charged ones like nucleic acid strands.^2^ The paradigm of the impermeability of the plasma membrane to large charged molecules was overturned only in 1988, when two reports came to light showing that the trans-activator of transcription (TAT) of the human immunodeficiency virus (HIV-1) could translocate across biomembranes.^3,4^ A few years later, Vivès, Brodin, and Lebleu demonstrated that a short fragment in the TAT structure, enriched in arginine and lysine, was sufficient to translocate across the cytosolic membrane and accumulate in the cell nucleus.^5^ Since its discovery, this small cluster and its derivatives have been successfully used to transport a variety of cargos into cells, including peptide nucleic acids (PNAs), siRNAs, plasmids, magnetic beads, and gene-editing proteins.^6^

Another relevant group of peptides capable of mediating the transport of exogenous molecules into cells are the nuclear localization signals (NLS).^7^ Shortly after the discoveries on TAT-HIV, the NLS with the sequence PKKKRKV from the simian virus 40 large T antigen (SV40T) was demonstrated to bind and internalize proteins larger than 60 kDa into the nuclei of rat liver cells.^8^ Over the years, peptides related to this NLS have been shown to be effective in facilitating the entry of molecules into the nucleus, as well as other important organelles such as the Golgi complex, lysosomes, and mitochondria in a variety of organisms.^9,10^ Along with a few other peptides reported in the 1990s,^11–14^ TAT-HIV and NLS-SV40T form a group of foundational molecules that inaugurated the field of cell-penetrating peptides (CPPs).^15–17^

Cationicity is a prevalent feature among CPPs, a characteristic they share with other bioactive peptides, including antimicrobial peptides (AMPs).^18,19^ Both TAT-HIV and NLS-SV40T, as well as most of the NLS identified to date,^20^ are almost exclusively composed of arginine and lysine residues. Interestingly, these molecules are often found in the proteomes of viruses capable of infecting primates^21,22^ and have a close relationship with transcriptional regulators of gene function.^23^ This frequent appearance in contexts related to folding and packing of genetic information hints at the usage of cationic CPPs to prepare peptiplexes – associative complexes formed between peptides and DNA – which offer a simple and cheaper platform for intracellular delivery of nucleic acids and are considered safer than viral vectors for gene therapy, albeit with some limitations in structural consistency and cellular uptake.^2^

In this study, we investigate and compare the structural dynamics of peptiplexes formed between DNA and the CPPs TAT-HIV and NLS-SV40T. Combining multiple experimental techniques and molecular dynamics (MD) simulations, we examine the structural features of these soft biomaterials at various scales. While both peptides are highly cationic, we observe that differences in the side chain chemistry of arginine and lysine strongly influence their interaction with nucleic acids, also affecting the supramolecular ordering of peptiplexes. Our findings show that TAT-HIV exhibits higher structural flexibility and self-assembles into a mixture of clusters and fibrillar arrangements upon DNA complexation, whereas NLS-SV40T keeps its native random-coiled structure, with peptiplexes forming globule-studded assemblies. Electrostatic and van der Waals forces, as well H-bonds stabilizing the arrangements, are critically affected by the peptide used in the complex, with TAT-HIV/DNA presenting stronger binding energies and predominance of H-bonds compared to NLS-SV40T/DNA. Both systems efficiently condense nucleic acids, leading to architectures in which DNA duplexes are very close to each other inside peptiplexes. However, NLS-SV40T/DNA displays a 2D hexagonal phase organization, which is hypothesized to correlate with the propensity of this peptide to settle into major grooves (thereby improving foldability), while TAT-HIV tends to bridge both handrails of the DNA staircase. Cell assays reveal that NLS-SV40T/DNA peptiplexes are imported more efficiently, an observation ascribed to its more compact organization and lower lytic potential. In contrast, TAT-HIV peptiplexes form membrane pores, as revealed by atomic force microscopy, suggesting that its stronger electrostatics and enhanced H-bonding capacity contribute to lytic activity. Our results, which encompass multiscale data obtained through both experimental and computational approaches, provide valuable insights into the structural landscape of peptiplexes derived from these archetypical CPPs and may assist the design of nanomaterials for nonviral delivery of nucleic acids.

## Materials and Methods

### Materials and Sample Preparation

No animal or human samples were used in this research, and all materials were acquired from commercial suppliers. Peptides were custom synthesized by AminoTech (São Paulo, Brazil) and delivered as TFA salts. The TAT-HIV and NLS-SV40T peptides, with sequences GRKKRRQRRRPPQ-NH_2_ (*M*_w_ = 1718 g/mol) and PKKKRKV-NH_2_ (*M*_w_ = 882 g/mol), respectively, were synthesized using standard solid-phase synthesis through the Fmoc strategy. From their amino acid composition, the liquid charges of the peptides are assumed to be +9 for TAT-HIV and +6 for NLS-SV40T at pH = 7. Liquid chromatography coupled with mass spectrometry assays indicated purities >98% for both peptides (Figures S1A and S2A). For HPLC analysis, mobile phase A was 0.1% TFA in water, and mobile phase B was 60% acetonitrile with 0.1% TFA in water. Aliquots of 20 μL of peptides at ∼ 1 mg/mL were injected into a Phenomenex C18 column, with elution monitored by UV/vis absorbance detector at λ = 214 nm. A gradient was run from 5% to 95% mobile phase B over 30 min for TAT-HIV and over 20 min for NLS-SV40T. The smoother elution gradient observed in the TAT-HIV run is a consequence of the higher cationicity of this peptide and its stronger ion-pairing with the TFA anion, as corroborated by the presence of tetra- and hexa-TFA adducts in the mass spectrum of this peptide (Figure S1B). Calf thymus DNA was obtained from Sigma-Aldrich. This DNA was composed of linear strands with polydisperse sizes in the range 10–20 kbp. The highly polymerized DNA was subjected to ultrasonication in a Diagenode Bioruptor according to methods described in previous works,^24^ and electrophoretic analyses indicated that the fragments obtained were mostly sized between 100 and 200 bp (Figure S3). The fragmented nucleotide strands were lyophilized. Given the polydispersity of the DNA strands, for all calculations throughout the work the average molecular weight of the base pair was used, Mw = 660. Solutions were prepared by weighing DNA or peptide powder in Eppendorf tubes and dissolving them in the appropriate buffer to the desired concentrations, which have been ascertained in the stocks via absorbance measurements at λ = 260 nm. Purity was ascertained by A_260_/A_280_ > 1.8. Samples were stored in the refrigerator for 3–5 days prior to characterization experiments involving spectroscopic, microscopy, or X-ray scattering techniques. At sufficiently high concentrations, the mixture of DNA and peptides led to the formation of visible white pellets which were analyzed by small-angle X-ray scattering (SAXS). For cell assays, 3-(4,5-dimethylthiazol-2-yl)-2,5-diphenyltetrazolium bromide (MTT) was also obtained from Sigma-Aldrich whereas reagents for culture medium preparation were obtained from Thermo Scientific.

### Circular Dichroism (CD)

CD measurements were performed using a Jasco J-810 instrument. The background spectrum of the cuvette containing buffer (PBS, 10 mM, pH = 7.2) was collected and automatically subtracted from each measurement. Complexes for CD assays were obtained by mixing DNA from a concentrated stock in a 1 mm CD cuvette containing peptides at concentrations of 36.5 μM TAT-HIV or 71 μM NLS-SV40T. The DNA amounts were selected to obtain complexes with different amine-to-phosphate ratios (N^+^/P^–^), covering formulations from an excess of positive charges to an excess of negative charges. The chosen charge ratios (N^+^/P^–^) were 2, 1, 0.66, and 0.5; therefore, the DNA concentrations varied from 82 to 330 μM in TAT-HIV complexes, and from 106 to 426 μM in NLS-SV40T/DNA mixtures. The DNA concentrations reported here and throughout this work correspond to the concentration of DNA base pairs in the final sample. Complementary measurements on peptide samples were conducted in 30% TFE to assess their helical capacity. In this case, to avoid buffer absorption effects in the range <200 nm, a shorter demountable cuvette (0.1 mm) was used, and the peptide concentrations were scaled accordingly. Data collection was performed at speed of 100 nm/min, with steps of 1 nm, and the resulting spectra were obtained from 5 accumulations. FFT filters (5-point windows) were used to smooth the data and eliminate random noise. For data analysis of complexes, difference spectra were calculated by subtracting the appropriately scaled spectrum of free DNA from the spectra of the peptide/DNA mixtures. The scaling factor accounted for the precise concentration of DNA in each complex. Secondary structure content was estimated using the DichroWeb online server,^25^ employing a reference data set optimized for denatured proteins.

### Isothermal Titration Calorimetry (ITC)

The heat flow resulting from the binding of the CPPs to DNA was ascertained using a VP-ITC microcalorimeter (MicroCal Inc., Northampton, MA). Given the absence of tryptophan or tyrosine in either TAT-HIV or NLS-SV40T compositions, peptide concentrations wre determined by measuring absorbance at 205 nm and calculating the corresponding molar absorptivities following the method proposed by Anthis and Clore (see details in Table S1).^26^ DNA concentrations were determined by using a Nanodrop apparatus. The reaction cell of 1.4576 mL in volume was filled with TAT-HIV (75 μM in 30 mM phosphate buffer, pH 7) or NLS-SV40T (65 μM in the same buffer). These concentrations values were chosen after exploratory measurements to define adequate signal-to-noise ratios. Then, one 1 μL aliquot followed by twenty-seven 10 μL aliquots of DNA (from a 1.4 mM stock in the same buffer) were injected stepwise, with a 600 s interval, into the working cell. The sample cell was constantly stirred (307 rpm), and the measurements were performed at 25 °C. Data was fit with the one-site binding model carried out with the analysis software provided by MicroCal.

### Small-Angle X-ray Scattering (SAXS)

SAXS measurements were conducted on the SAXS-1 beamline at LNLS (Campinas, Brazil). Peptiplex samples were prepared by mixing appropriate amounts of peptides and DNA to attain an amine-to-phosphate ratio N^+^/P^–^ = 2. In the case of SAXS measurements, final peptide concentrations were set at 1 mM for both peptides. The corresponding DNA concentrations were 2.25 and 1.5 mM for complexes prepared with TAT-HIV and NLS-SV40T, respectively. The use of larger concentrations in SAXS measurements is dictated by the sensitivity of the method, which requires concentrations in the millimolar range for short peptides to achieve adequate signal-to-noise ratios. This procedure led to the formation of white pellets which were sandwiched in-between mica foils into a 1 mm path length cell. Samples were kept at 25 °C using a water circulation system coupled to the sample holder. Data were collected in 10 frames of 30 s each and, in the absence of radiation damage, the frames were averaged and background subtracted. The energy of the X-rays was set to 8 keV (λ = 1.54 Å), and the sample-to-detector distance was determined to be at 893 mm using silver behenate as a standard. This configuration gave access to *q*-vectors in the range 0.12 nm^–1^ < *q* < 4 nm^–1^, roughly corresponding to length scales situated between 1.5 and 50 nm.

### Transmission Electron Microscopy (TEM)

TEM imaging was performed with a JEOL-2100 FEG-TEM instrument at LNNano, Campinas, Brazil. Lacey carbon grids (300 mesh) were subjected to glow discharge prior to sample deposition. Complexes were prepared at an N^+^/P^–^ = 2. TAT-HIV/DNA mixtures were formulated with 290 μM peptide and 652 μM DNA. NLS-SV40T/DNA complexes were prepared with 570 μM peptide and 855 μM DNA. Control samples containing only peptides were prepared at twice these concentrations (580 μM for TAT-HIV and 1140 μM for NLS-SV40T) without DNA. The relatively high concentrations in TEM experiments were necessary to provide sufficient particle density and contrast on the grids. This led to the formation of pellets that remained suspended in the solution. A 3 μL volume of sample was applied to each grid and allowed to rest for 60 s at room temperature. Excess solution was removed with filter paper, and counterstaining was performed by depositing 5 μL of 2% uranyl acetate solution for 30 s, followed by additional staining with 2% uranyl acetate and removal of excess solution with filter paper. The microscope operated at 200 keV, and data was further analyzed using ImageJ.

### Atomic Force Microscopy (AFM)

AFM imaging of peptiplexes was carried out using a Nanosurf FlexAFM instrument operating in dynamic (tapping) mode. Samples were prepared by depositing droplets from peptiplex solutions on freshly cleaved mica substrates, which were left to dry overnight in desiccators. Both peptiplexes were prepared at N^+^/P^–^= 2, with TAT-HIV/DNA complexes formulated with 60 μM peptide and 135 μM DNA, and NLS-SV40T/DNA peptiplexes prepared with 100 μM peptide and 150 μM DNA base pairs. The cantilevers used in the experiments had a nominal force constant of 40 N/m and a resonance frequency of 325 kHz. The tip free vibration voltage was set at 2 V, with a set point at 55%, and a scanning frequency of 0.5 Hz. Typical PID gains were P = 550, I = 1000, and D = 0. Data from fixed cells incubated with peptiplexes were acquired using a Park XE7 instrument also operating in dynamic mode (cantilever force constant = 5 N/m, resonance frequency = 160 kHz). We carried out AFM topography imaging of cells incubated with high concentrations of peptiplexes to analyze damage induced by peptiplexes. Cell culture conditions are detailed below alongside fluorescence microscopy procedures. For these AFM assays, HeLa cells were incubated for 4 h with peptiplexes formulated at N^+^/P^–^= 2 using a high peptide concentration of 100 μg/mL (equivalent to 58.2 μM TAT-HIV + 130 μM DNA or 113.4 μM NLS-SV40T + 170 μM DNA). Fixation was performed using standard approaches with 4% paraformaldehyde (PFA) as reported elsewhere.^27,28^ Image flattening and enhancement were conducted using Gwyddion software.^29^

### Molecular Dynamics (MD) Simulations

All-atom model build-up as well as short (<40 ns) MD simulations and analyses were conducted using the Amber24 software,^30–32^ while long (≥100 ns) simulations and analyses were conducted using the GROMACS version 2025.3 package.^33^ A 22-bp B-form DNA duplex with alternating guanine (G) and cytosine (C) bases, i.e., (GC)_11_ or 5′-d­(GCGCG­CGCGCG)-3′ with the net charge of −44|e|, was constructed using the TLEAP module in the AmberTools24.^31,32^ Two peptides, TAT-HIV and NLS-SV40T, were generated with the TLEAP module, based on their amino acid sequences and C-terminus capping schemes used in the experiments (GRKKRRQRRRPPQ-NH_2_ and PKKKRKV-NH_2_). The net charges were confirmed to be +9|e| and +6|e|, respectively, from their uncapped N-terminus and basic amino acids, arginine (R) and lysine (K). The OL21 force field (FF) was employed for DNA,^34^ and the ff19SB FF was used for peptides,^35^ since they are extensively validated and widely recommended state-of-the-art nonpolarizable FF’s that offer a strong balance between physical accuracy and computational tractability for DNA–protein systems.^35–40^ Polarizable FF’s may provide a more accurate description of DNA–protein binding, but they still remain computationally prohibitive for the extensive sampling required here. The net charges of the DNA (−44|e|), TAT-HIV (+9|e|), and NLS-SV40T (+6|e|) were neutralized by adding counterions (44 Na^+^, 9 Cl^–^, and 6 Cl^–^, respectively) at random positions around each system, using the 12–6–4 Lennard-Jones ion model developed by Li and co-workers.^41^ A total of 12 distinct initial configurations of each peptide around a single DNA duplex were generated in PDB format, using the PACKMOL software,^42^ as described in detail in the Supporting Information (SI) around Table S4 and Figure S10. Each peptiplex configuration was solvated in a cubic periodic box of 9.0 nm on each side by adding 14,918 and 21,878 water molecules to the TAT-HIV/DNA and the NLS-SV40T/DNA system, respectively. The water molecules were described by the OPC water model,^43^ which has provided good structural descriptions of DNA and proteins when combined with the OL21 and ff19SB FF’s.^35,36^ The long-range electrostatic interaction in the periodic boundary condition was treated using the particle-mesh Ewald (PME) method.^44^ A summary of all the system details can be found in Tables S4–S7 of SI. The initial simulation box was chosen to be less dense than 1 g cm^–3^ in order to allow facile approach between DNA and peptide. During the simulations, the box sizes were naturally reduced to 7.7 nm. Each periodic system was submitted to energy minimization using the SANDER module of Amber24 and then gradually heated from 0 to 300 K over 20 ps MD simulations under constant volume (NVT) conditions, followed by 14 ∼ 29 ns of equilibration and 20–35 ns of production MD simulations under constant pressure (NPT) conditions at 300 K and 1 atm, using the PMEMD module of Amber24.^32^ These short simulations were followed by longer (up to 600 ns) NPT runs using the GROMACS package.^33^ The temperature control was achieved by the Langevin (Amber) and V-rescale (GROMACS) thermostats, while pressure was regulated with the Berendsen (Amber) and Parrinello–Rahman (GROMACS) barostats.^32,33^ All the covalent bonds involving H atoms were constrained by the SHAKE (Amber) and LINCS (GROMACS) algorithms,^32,33^ allowing 2 fs of time step for integration. The simulations were analyzed over the last 20 ns of production runs, using the CPPTRAJ software in AmberTools24 and the GROMACS in-built analysis tools.^32,33,45^ The radius of gyration (*R*_g_) of each peptide, free and DNA-bound, was determined to monitor the structural characteristics. The secondary structures of both peptides were analyzed using the Ramachandran plots^46^ and the DSSP (Dictionary of Secondary Structure in Proteins) analysis.^47,48^ The intermolecular interaction between DNA and each peptide was analyzed using linear interaction energy profiles, intermolecular hydrogen (H) bond profiles and maps, intermolecular contact maps,^32,49^ and MMPBSA (Molecular Mechanics Poison Boltzmann Surface Area) analyses.^32,45,50^

### Cell Culture and Fluorescence Imaging on Confocal Microscope

HeLa cells were obtained from ATCC and cultured in Dulbecco’s Modified Eagle Medium (DMEM) supplemented with 10% fetal bovine serum, penicillinstreptomycin, and 2 mM glutamine (ThermoFisher) and maintained in a humidified cell-incubator at 37 °C with 5% CO_2_. In each well of a 24-well plate, a sterilized glass coverslip was placed at the bottom, and 5 × 10^4^ cells were seeded and incubated for 24 h under the same conditions. After incubation, the cells were washed three times with PBS to remove excess serum and cell debris. DNA stocks were previously prepared at 0.1 mg/mL (150 μM) in sterile water. NLS-SV40T/DNA peptiplexes were prepared using DNA labeled with the fluorescent probe YOYO-1 (Thermo Scientific). To formulate complexes, 50 μL of the DNA stock were transferred to sterile test tubes, totaling 5 μg of DNA in each tube. Peptide was added from a stock in a total mass of 4.5 μg to match N^+^/P^–^ = 2, a condition frequently reported in the literature to enhance the cellular internalization of nucleic acid complexes.^15,51^ The tubes were left at rest for about 30 min for complexation and then their contents were transferred to wells containing DMEM without serum, adjusted to a final volume of 1 mL. Under these conditions, the final molar concentrations in contact with cells during uptake assays were 5.1 μM peptide and 7.6 μM DNA (base pairs). Cells were incubated with peptiplexes for 4 h prior to analysis. Subsequently, the cells were washed three times with PBS to remove unadhered cells and unbound complexes. Adherent cells were fixed with 4% paraformaldehyde and stained with 5 μg/mL DAPI (4′,6-diamidino-2-phenylindole, Thermo Scientific) in PBS for 5 min to visualize cell nuclei. After staining, the cells were washed three times with PBS and imaged using a confocal microscope (Leica TCS SP8, Mannheim, Germany).

### MTT

Peptiplexes for MTT assays were prepared following the same procedure described for cellular uptake assays, but with varying peptide amounts covering a range of charge ratios (N^+^/P^–^ ∼ 0.1–1.8). Briefly, 5 μg of DNA was transferred to sterile test tubes, followed by the addition of varying amounts of peptide. The final DNA concentration was fixed at 7.6 μM (base pairs), while peptide concentrations ranged from 0.15 to 2.3 μM for TAT-HIV and 0.3 to 4.5 μM for NLS-SV40T, corresponding to mass concentrations of 0.25 to 4 μg/mL for both peptides. Details on the composition of each peptiplex formulation are displayed in Tables S2 and S3. HeLa cells were seeded in 96 well plates with a density of 4 × 10^3^ cells per well in supplemented DMEM and incubated overnight under controlled temperature and 5% CO_2_ atmosphere. After this step, cells were washed 3 times with PBS to remove the medium; in the following, they were incubated in 100 μL supplemented DMEM containing DNA or peptiplexes. After 72 h of incubation, the cells were washed again 3 times with PBS prior to the addition of 100 μL of DMEM containing 0.5 mg/mL MTT. The plates were then incubated for 4 h in the dark and 100 μL DMSO was added to the wells to dissolve the resulting formazan crystals produced due to MTT reduction in cell mitochondria. The plate was gently agitated at 37 °C for 15 min, and absorbance of each well was measured at 570 nm using a SpectraMax M2 microplate spectrophotometer (Molecular Devices, San Jose, USA). Data analysis from triplicates were plotted and analyzed using GraphPad Prism software (GraphPad Software, USA). Significance was assessed by comparing the means and standard deviations of controls and peptide-incubated groups using Bonferroni-corrected Welch tests.

## Results

### Secondary Structure and Thermodynamics of Complexation

We began our structural characterization by investigating the secondary structure of TAT-HIV and NLS-SV40T using circular dichroism (CD). Figure 1A and B presents CD spectra of peptide solutions and their corresponding peptiplexes formulated at charge ratios (N^+^/P^–^) ranging from 0.5 to 2, encompassing formulations from an excess of anionic charges to an excess of cationic charges. The data from peptide solutions (black curves in Figure 1) reveal that both peptides present spectra featured by a strong minima at ∼ 198 nm, traditionally associated with n→π* transitions in the peptide bonds.^52^ This spectral signature suggests a large fraction of unordered chains in the samples, a conclusion further supported by the hyperbolic profile observed in the Kratky plots of the SAXS data from the peptide solutions (Figure S4). These spectral features could also indicate the presence of polyproline II (PPII) conformations,^53^ in line with findings obtained in MD runs (see below). Although the CD spectra of both TAT-HIV and NLS-SV40T are characterized by an intense minimum characteristic of disordered conformations, the band is noticeably broader for TAT-HIV than for NLS-SV40T. This shape of the spectrum suggests that the TAT-HIV peptide is allowed to assume a wider range of dihedral angles, indicating a higher degree of conformational heterogeneity compared to the NLS-SV40T peptide. This interpretation aligns with either MD simulations (see below) or estimations performed using the DichroWeb server, which indicated higher difficult to perform data fitting for TAT-HIV compared to NLS-SV40T (Figure S5). These results, however, should be considered with caution due to the suboptimal goodness of the fits arising from the inherent limitations of reference data sets within DichroWeb for accurately analyzing the CD spectra of short peptides.^25^ Unfortunately, the CD spectra of random coils and PPII helices are quite similar and difficult to distinguish unequivocally.^53,54^ However, the presence of weak positive bands near 220 nm has been interpreted as potentially accounting for PPII conformations;^55^ in our case, this feature is more pronounced in samples containing the NLS-SV40T peptide. Complementary measurements performed in the presence of trifluoroethanol (TFE), a solvent widely known to induce order in peptides,^56^ further reveal positive bands near 215–220 nm, consistent with increased ordering (Figure S6).^53^ Therefore, this initial assessment of the secondary structure indicates that both peptides are predominantly disordered, with TAT-HIV showing higher conformational heterogeneity.

**Figure 1:**
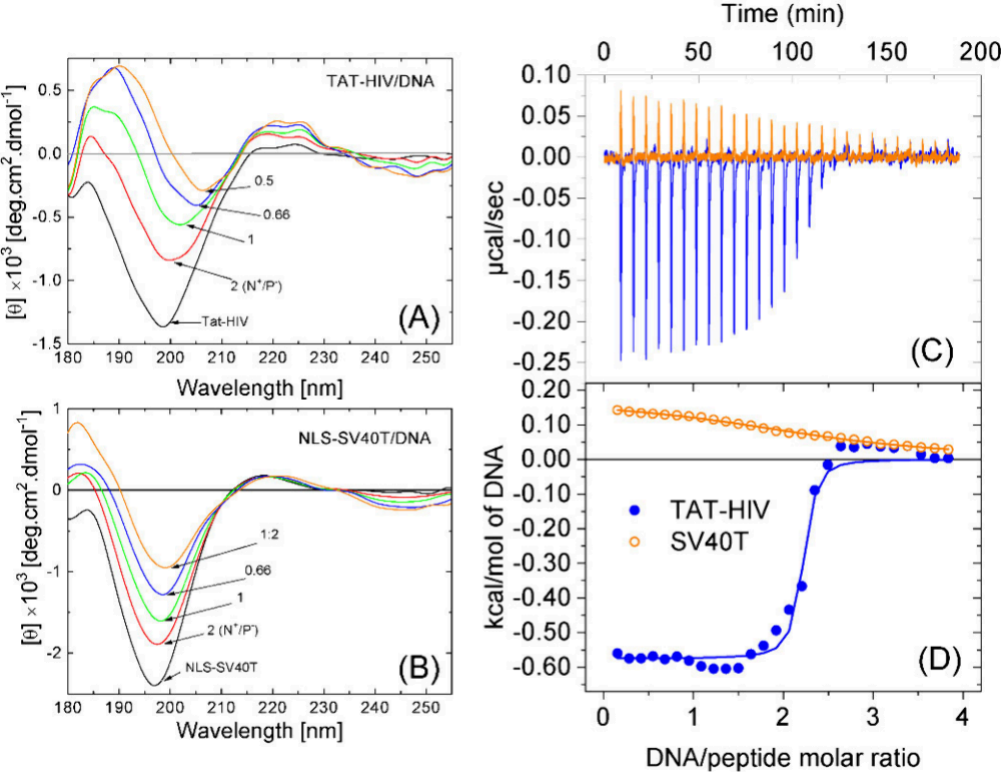
(A) CD data from peptiplexes prepared with TAT-HIV and (B) NLS-SV40T at different N^+^/P^–^ ratios. (C) Heat flow over time from CPP solutions (75 μM TAT-HIV or 65 μM NLS-SV40T) titrated with DNA fragments from a 1.4 mM base pair stock solution. (D) Integrated heat per mole of DNA base pairs as a function of the DNA base pairs/peptide molar ratio. The solid lines correspond to fits with the one-site model.

Upon DNA complexation, however, secondary structures evolve differently according to the peptide used in the formulation of peptiplexes. While TAT-HIV/DNA peptiplexes exhibit a more pronounced redshift of the negative band from ∼198 nm to ∼208 nm along with a redshift of the 185 nm signal to ∼190 nm (Figure 1A), NLS-SV40T retains its random coil signature with a reduction in negative signal intensity and the n→π* transition marginally displacing from 197 to 199 nm (Figure 1B). The distinctions in the structural evolution of the complexes are more pronounced in CD difference spectra, obtained after subtracting the DNA contribution from the complex spectrum (see Figure S7). In this case, one observes that the effect over the negative band is even greater in TAT-HIV/DNA peptiplexes, in which the spectral broadening again indicates higher conformational variability. Notably, these difference spectra reveal the emergence of a negative band near 220 nm for both peptides, which is consistent with the decrease of disordered fractions and growth of ordered conformations in both peptiplexes. We performed estimations of the secondary structure content on the CD difference spectra of the complexes using DichroWeb. Given that its data sets are optimized for proteins and struggle to describe data from short peptides, whose spectra are strongly biased by side chain transitions, it was only possible to tentatively analyze data from formulations containing lower DNA content, at N^+^/P^–^ = 2. These analyses suggested the presence of only minor α-helice contents in both peptiplexes, with larger fractions of sheets and turns. However, these semiquantitative estimations, which are inherently limited for short peptides, returned fits with suboptimal quality, preventing unequivocal structural quantification. Despite of this, the analysis consistently indicated a more pronounced reduction in disordered content upon association with DNA, especially for NLS-SV40T. Overall, the CD data indicate distinctions in peptide-DNA interactions, with TAT-HIV peptiplexes showing a more intricate secondary structure compared with NLS-SV40T peptiplexes.

We further evaluated peptide-DNA interactions by performing isothermal titration calorimetry (ITC) to obtain thermodynamic information on peptiplexes. Representative thermodynamic data are shown in Figure 1C and D. In both cases, the one-site binding model^57^ could reasonably well fit the data (see lines in Figure 1D) with the parameters listed in Table 1. It is important to mention that these parameters could represent an effective total interaction, which entail contributions from different processes, such as electrostatic interaction, hydrogen bonding and counterion release. The results indicate that the association between TAT-HIV and DNA is markedly exothermic, with ΔH ∼ −0.6 kcal/mol of DNA base pair, characterized by a stoichiometry factor *n* ∼ 2 and an association constant at *K* ∼ 6 × 10^6^ M^–1^. We can use *K* to obtain ΔG and TΔS from fundamental thermodynamic relations as ΔG ∼ −9.3 kcal/mol and TΔS ∼ 8.7 kcal/mol. These values for *K* and ΔG are in close agreement with previous measurements.^58^ In contrast, for NLS-SV40T/DNA samples, ITC data were featured by weaker endothermic signals (ΔH ∼ 0.2 kcal/mol) with a slow-decaying profile, consistent with a lower affinity (orange curves in Figure 1C and D). Fitting of this curve revealed a slightly higher stoichiometry at *n* ∼ 2.5, but with an association constant 2 orders of magnitude lower (K ∼ 3 × 10^4^ M^–1^), from which the other thermodynamic variables can be obtained as ΔG ∼ −6.1 kcal/mol and as TΔS ∼ 6.3 kcal/mol.

**Table 1:** Summary of Thermodynamic Parameters Derived from ITC Data Fitting to the One-Site Binding Model

Peptiplex	*n*	K [M^–1^]	ΔH [kcal/mol]	ΔS [cal/K·mol]	ΔG [kcal/mol]
TAT-HIV/DNA	2.2	6.3 × 10^6^	–0.58	29	–9.3
NLS-SV40T/DNA	2.5	2.8 × 10^4^	0.17	21	–6.1

Putting the findings above together, we conclude that in both cases association is driven mainly by entropy. This conclusion is consistent with previous studies which proposed that the formation of peptiplexes relies on the entropic gain originating from the counterion release and desolvation of biomacromolecules.^27,59,60^ It should be mentioned that all thermodynamic variables were calculated per mol of base pairs, and a different scenario would be obtained if the data were given for the whole DNA sequence. However, the interaction of ligands with DNA quite often occurs locally with the grooves of the DNA double helix strand, irrespective of the size of the whole DNA molecule. Thus, the interaction is not with the DNA as a single entity, but rather with the base pairs. In fact, the interaction of ligands with DNA is quite always treated at the base pair level in the literature.^58,61^ The fact that the enthalpy variation is exothermic for TAT-HIV and endothermic for NLS-SV40T reveals that, although both peptides are strongly cationic, their association with nucleic acids is very distinct in each case, with peptide-DNA association being markedly more favorable for TAT-HIV and entropy dependence even more preponderant for NLS-SV40T. The different association behavior of these peptides with nucleic acids was visually confirmed by gel electrophoresis (see Figure S8), which revealed strong DNA condensation by TAT-HIV below charge neutrality (N^+^/P^–^ ≤ 1), while NLS-SV40T showed partial complexation even at a 2:1 charge ratio. This direct visualization of weaker association in NLS-SV40T/DNA peptiplexes is consistent with the ITC and CD data, and it highlights that the interaction is sequence-dependent and not exclusively driven by electrostatic balance.

### Polymorphism and Supramolecular Structure

The next step in our analysis was to evaluate the nanoscopic structure of the peptiplexes via small-angle X-ray scattering (SAXS). Scattering profiles from samples containing only peptides or nucleic acids do not show characteristics of supramolecular ordering (SI file, Figure S5). Figure 2 shows SAXS data from DNA complexes prepared with TAT-HIV or NLS-SV40T (at a 2:1 amine-to-phosphate ratio), indicating that the supramolecular organization is highly dependent on the peptide used in the formulation. The *q*-range used in the analyses carries information from length scales roughly ranging from 1 to 50 nm, covering dimensions corresponding to a few complexed strands up to the level of small peptide/DNA aggregates.

**Figure 2:**
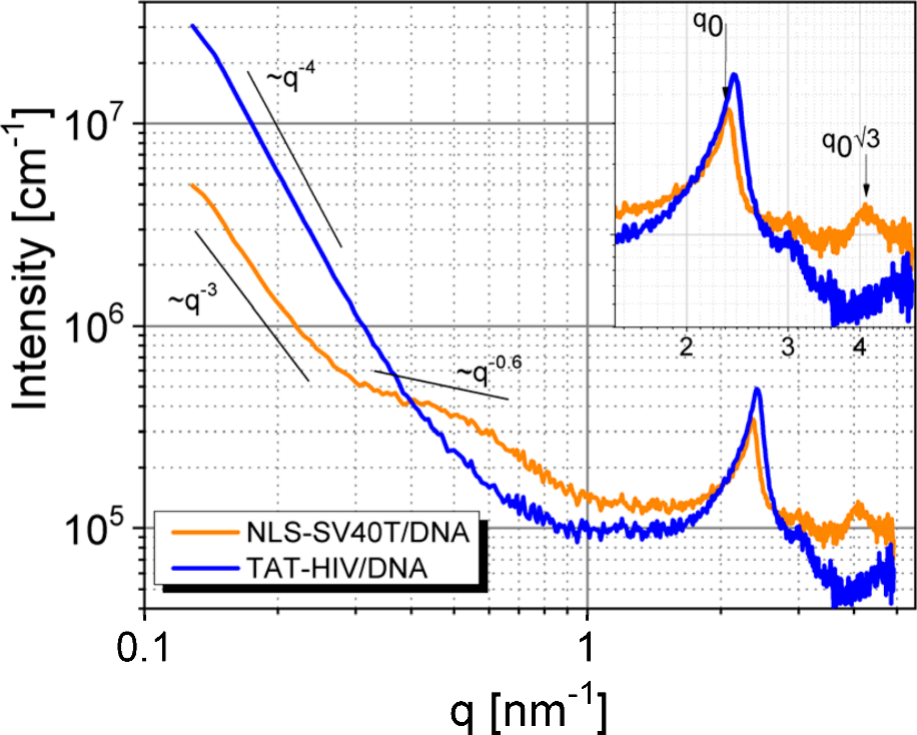
SAXS data from peptiplexes prepared at 2:1 ratio (N^+^:P^–^). The inset shows details of Bragg peaks emerging at the high-*q* region, including the √3 × *q*_0_ peak, corresponding to the *q*_11_ reflection of a 2D hexagonal phase formed by DNA strands.

To facilitate comparison between peptiplexes, we divided the *q*-range into three main regions. In the low-angle region, 0.13 < *q* < 0.25 nm^–1^, associated to sizes approximately between 25 and 50 nm, the scattering is characterized by a linear decay in the log–log representation. This behavior, with a scaling law describing the data, can be attributed to the presence of surface fractals in both peptiplexes. The nature of the fractals differs between peptides. While TAT-HIV/DNA complexes exhibit a power law behavior scaling with ∼*q* ^–4^, characteristic of sharp interfaces,^62,63^ peptiplexes prepared with NLS-SV40T scale with ∼*q* ^–3^ in the low-angle region, indicative of diffuse surfaces.^64^

The intermediate *q* region, 0.25 < *q* < 1.5 nm^–1^, associated with length scales between approximately 4 and 20 nm, also reveals substantial differences between TAT-HIV and NLS-SV40T. In this range, scattering data from TAT-HIV-based samples maintain the power law decay with an exponent of −4, indicating that the presence of surface fractals also extends to the range of several nanometers. In contrast, for peptiplexes formulated with NLS-SV40T, the intermediate *q* region exhibits a plateau scaling with ∼*q* ^–0.6^, which can be attributed to the presence of globular inhomogeneities within the complexes.^65^

Finally, in the high-*q* region, *q* > 1.5 nm^–1^, the data carry information on the local structure at the level of individual molecular components. In both peptiplexes, this region is dominated by a strong Bragg peak indicating a high degree of order within complexes. In the case of TAT-HIV-based peptiplexes, this peak appears at *q*_00_ = 2.41 nm^–1^, while for complexes prepared with NLS-SV40T, it is located at *q*_00_ = 2.36 nm^–1^. These peak positions correspond to repeats of 2.60 nm, for TAT-HIV/DNA, and a slightly larger value of 2.66 nm for NLS-SV40T peptiplexes. A relevant feature is the emergence of a second peak at *q* = 4.08 nm^–1^ in NLS-SV40T/DNA samples. This peak is located at a position equivalent to √3 × *q*_00_, suggesting that it corresponds to the *q*_11_ reflection of a 2D hexagonal columnar phase.^66^ In this case, the lattice parameter can be estimated at *a* = 4π/(√3 × *q*_11_) = 3.07 nm, in close agreement with previous measurements of units cells in polylysine/DNA complexes which indicated *a* = 3.04 nm.^67^ The sizes derived from SAXS data are comparable to the diameter of hydrated B-DNA chains, which is around 2.4 nm.^68^ In summary, the SAXS data demonstrate that the internal structure of both complexes is very compact, with the DNA chains positioned very close to each other.

The SAXS measurements described above were complemented by observations from electron microscopy, which provided visualizations of the peptiplexes in real space. Figure 3 displays micrographs obtained from either peptide solutions or TAT-HIV/DNA and NLS-SV40T/DNA complexes, prepared at a 2:1 charge ratio. The images reveal that at the concentration used in the peptiplexes, 1 mg/mL, neither TAT-HIV nor NLS-SV40T are capable of forming aggregates on their own, consistent with the high solubility of these species (Figure 3A and C). Similar to the SAXS observations, we found that when combined with DNA, the resulting peptiplexes exhibit quite distinct morphological aspects depending on the peptide used in the formulation. In the case of NLS-SV40T peptiplexes, shown in Figure 3B, we observed self-organization in the form of globular polymorphs with diameters around 20 nm. In addition, it is possible to distinguish the association of some of these structures to form more elongated arrangements with lengths that reach ∼60 nm. This finding can be correlated with the highly compact structure revealed by SAXS data above, which showed the presence of surface fractals with diffuse interfaces and an inner structure characterized by highly condensed DNA chains forming hexagonal phases. In the case of peptiplexes prepared with TAT-HIV, Figure 3D, we observed the coexistence of prolate aggregates, with dimensions exceeding 100 nm along their longest axis, and fibers with thicknesses around 5 nm and lengths of several hundred nanometers. The coexisting fibers are very flexible as indicated by the extensive presence of bends and twists along their length. The presence of large aggregates is consistent with the SAXS data, which indicated the presence of surface fractals with sharp interfaces. AFM topography images from peptiplexes solutions dried on mica substrates further supported that the propensity of TAT-HIV/DNA to form elongated clusters and the tendency of NLS-SV40T/DNA to fold into globular arrangements likely extends to higher length scales and concentrations (SI file, ).

**Figure 3:**
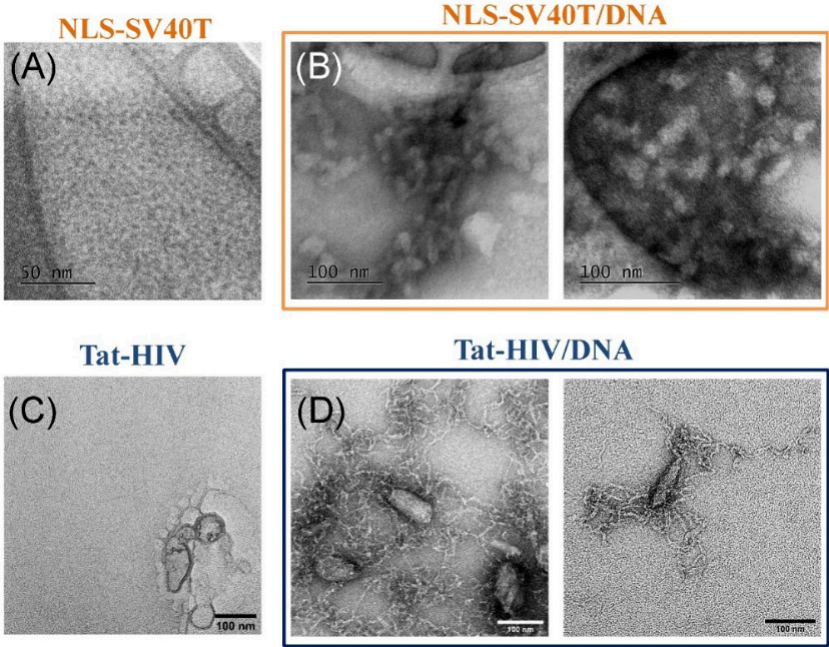
Electron micrographs from peptiplexes prepared with the NLS-SV40T (top row) and TAT-HIV (bottom row). Control Images from peptide solutions are shown in (A) and (C), while peptiplexes prepared at 2:1 amine-to-phosphate ratio are shown in (B) and (D).

### MD Simulations

To provide further insights into the association between CPPs and DNA, we performed MD simulations to examine the effect of the peptide composition on DNA binding energetics, residue-level interactions, and secondary structure stability. Our findings illustrate the distinct modes of DNA engagement exhibited by arginine (R)-rich and lysine (K)-rich peptides.^49^ Figure 4 shows the molecular models used in the simulation, DNA modeled as a 22-bp B-form DNA duplex (GC)_11_ as well as the TAT-HIV and NLS-SV40T peptides with arginine and lysine residues highlighted in blue and red, respectively.

**Figure 4:**
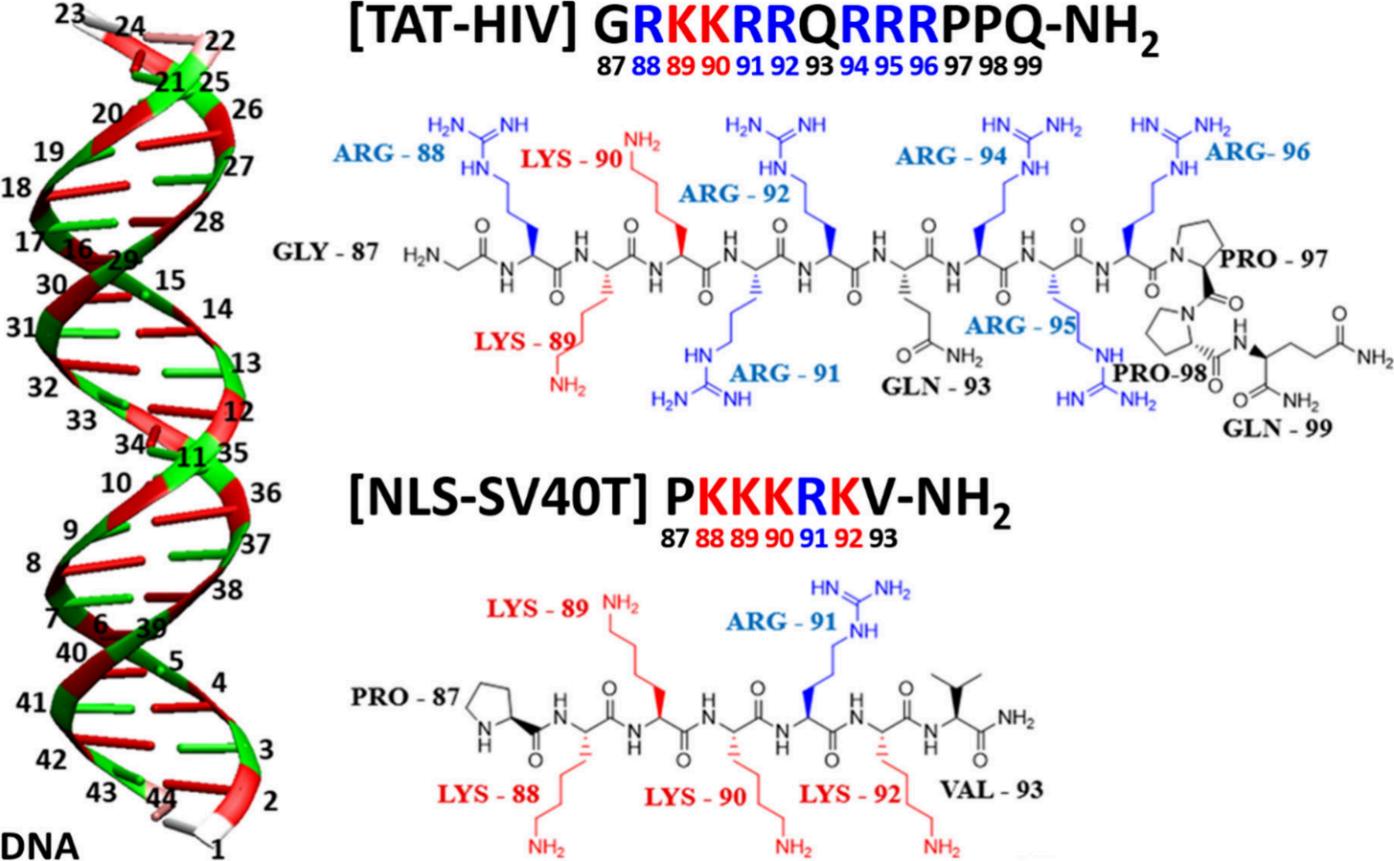
(Left) 22-bp DNA duplex model (GC)_11_. (Right) TAT-HIV and NLS-SV40T, cationic cell-penetrating peptides (CPPs), composed of Arg (R), Gln (Q), Lys (K), Gly (G), Pro (P), and Val (V). The numbering convention is used below to analyze residue-level interaction (see Figure 6).

Extensive simulations started from the 12 distinct initial configurations of each peptide around a DNA duplex produced essentially the same type of binding configurations (Figures S10–S12, SI). Therefore, we herein present the results analyzed on the final 20 ns of the longest (600 ns) MD simulations. Their intermediate and final snapshots are shown in Figure 5.

**Figure 5:**
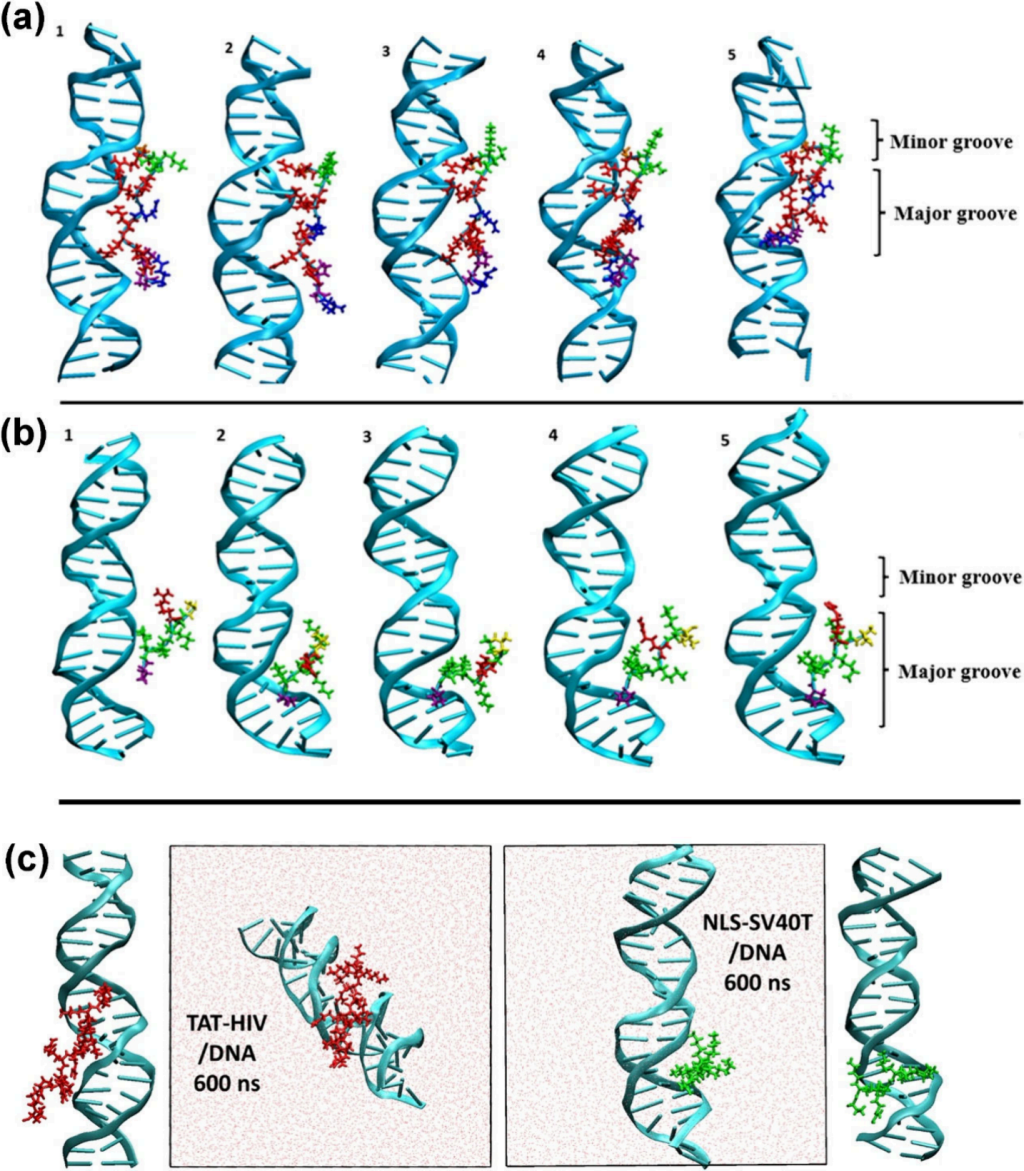
Snapshots of TAT-HIV/DNA and NLS-SV40T/DNA, with or without water and counterions and viewed from different directions, (a, b) taken at five lowest local minima of the DNA–protein intermolecular interactions during the first 34 ns NPT simulations and (c) after the whole 600 ns NPT simulations, whose final 20 ns trajectories are shown as Videos S1, S2, and S3 in the SI. Color code: (GC)_11_ DNA (cyan), TAT-HIV (red), NLS-SV40T (green), water (light red), Arg (R; red), Lys (K; green), Pro (P; purple), Gly (G; orange), Gln (Q; blue), and Val (V; yellow).

NLS-SV40T, due to its shorter length, lower positive charge, and the small and aliphatic ammonium end groups of the predominant Lys (K) residues (Figure 4), exhibits more dynamic movement along the DNA in the early stage of the simulation (compare SI files, movies 1, 2 and 3), but eventually settles into and remains bound within the major groove of the DNA (Figure 6b). We can hypothesize that a number of NLS-SV40T peptides would bind along the pocket of the DNA major groove to form a compact and structurally consistent charge-neutral NLS-SV40T/DNA complex. We can also propose that self-assembly of such compact and rigid NLS-SV40T/DNA complexes would lead to a 2D hexagonal organization, as observed in the experiments.

**Figure 6:**
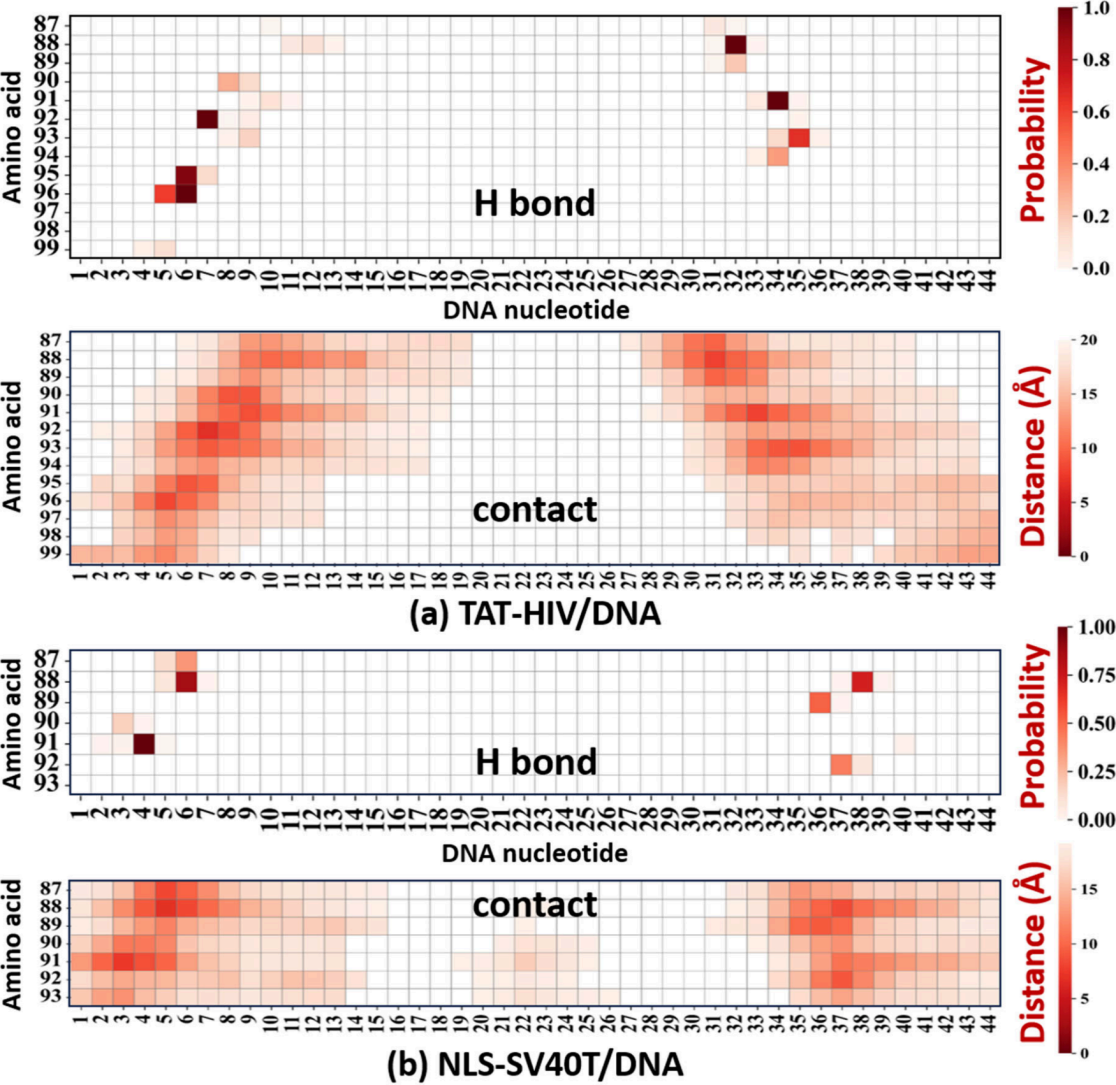
Heatmaps showing residue-specific intermolecular interaction between DNA nucleotides (*x*-axis) and amino acids (*y*-axis) of (a) TAT-HIV or (b) NLS-SV40T in water, which were analyzed over the last 20 ns of the 600 ns NPT simulations at 300 K. The pixel darkness reflects the probability of H-bond formation (top panels) and the contact distance defined as the shortest average distance (in Å) between atom pairs of the amino acid and the nucleotide (bottom panels). The numbering of DNA nucleotides and peptide amino acids are given in Figure 4 above. The total H-bond counts are shown in Figure S14.

On the other hand, TAT-HIV is unable to fit into any single DNA groove (Figure 4), probably due to its length, large volume, and the bulky, planar, π-delocalized, and π-stack-inducing (*acyclic aromatic*) guanidinium end groups of the predominant Arg (R) residues. Instead, TAT-HIV eventually binds simultaneously to the major and minor grooves through its two terminal residues (Figure 6a), while its central domain remains mostly outside the grooves, positioning itself to bridge two DNA duplexes. Similar characteristics, multiple-point binding of TAT-HIV to DNA, have also been demonstrated for the protamine^69–72^ and its R-rich short model (RRRSRRRS).^73^ We thus expect that, while TAT-HIV binds more strongly to DNA than NLS-SV40T, it may lead to a less compact and less ordered DNA self-assembled organization than NLS-SV40T.

#### MMGBSA Binding Energy

As a quick step to determine which peptide-DNA complex has a stronger binding, their approximate binding energies in water (Δ*E*_bind,aq_) were estimated with the MMGBSA (Molecular Mechanics Generalized Born Surface Area) method. Table 2 shows that TAT-HIV/DNA binding is more favorable than that of NLS-SV40T/DNA (Δ*E*_bind,aq_ = −68.9 vs −45.1 kcal/mol). It is interesting to see that the difference between them (ΔΔ*E*_bind,aq_ = 23.5 kcal/mol) comes mostly from the difference in the nonelectrostatic interaction (ΔΔ*E*_vdw,g_ = 16.0 kcal/mol), while their binding is mostly electrostatically driven (Δ*E*_elec,g_ and Δ*E*_GB,desolv_). The strong electrostatic attraction between negatively charged DNA and positively charged peptides (Δ*E*_elec,g_ = −639.4 and −430.2 kcal/mol) is offset by the strong desolvation penalty upon binding (Δ*E*_GB,desolv_ = 621.3 and 417.7 kcal/mol). The net electrostatic interaction after this offset (−18.1 vs −12.5 kcal/mol) is also more favorable for TAT-HIV/DNA than for NLS-SV40T/DNA. In summary, TAT-HIV/DNA binding is stronger than NLS-SV40T/DNA binding, probably owing to stronger dispersive attraction made by the guanidinium terminal groups of the Arg (R) residues dominant in TAT-HIV as well as the stronger positive charge (+9|e|) of TAT-HIV.

**Table 2:** DNA-Peptide Binding Energy in Water by MMGBSA^a^

Energy components (kcal/mol)	TAT-HIV/DNA	NLS-SV40T/DNA
(1) Δ*E*_vdw,g_	–44.0 ± 0.1	–27.99 ± 0.07
(2) Δ*E*_elec,g_	–639.4 ± 0.2	–430.2 ± 0.1
(3) Δ*E*_bind,g_ (= 1 + 2)	–683.4	–458.2
(4) Δ*E*_GB,desolv_	621.3 ± 0.2	417.7 ± 0.1
(5) Δ*E*_surf,desolv_	–6.79 ± 0.01	–4.63 ± 0.01
(6) Δ*E*_desolv_ (= 4 + 5)	614.5	413.1
(7) Δ*E*_bind,aq_ (= 3 + 6)	–68.9	–45.1

a Analyzed over the last 20 ns of the 600 ns NPT simulations at 300 K.

Moreover, a number of Na^+^ ions expelled from DNA during the peptide binding would contribute to an entropy gain and in turn to a more favorable binding free energy. We expect that this effect would be larger for longer and more-cationic TAT-HIV than for NLS-SV40T. Indeed, the radial distribution function (RDF) and the coordination number (CN) of Na^+^ ions around each phosphate O atom in DNA (Figure S13, SI) are lower in the TAT-HIV/DNA complex than in the NLS-SV40T/DNA complex, indicating a greater degree of Na^+^ ion expulsion by TAT-HIV. These results are in line with the findings of the calorimetric experiments shown above in Table 1.

#### Site-Specific H-Bond/Contact Maps

Such differences in binding characteristics of the two peptides are also supported by the difference in the total number of H-bonds with DNA (∼5 for NLS-SV40T and ∼10 for TAT-HIV; Figure S14, SI). Furthermore, site-specific (or residue-level) intermolecular H-bond and contact maps (Figure 6, top and bottom) indicate the strength of interaction between each pair of peptide amino acid (*y* axis) and DNA nucleotide (including base, sugar, and phosphate; *x* axis) [see Figure 4 for their identification]. The darkness of each pixel in the site-specific protein–DNA H-bond maps (Figure 7a-b, top panels) indicates the probability of forming residue-specific H bonds, i.e., the fraction of simulation frames where specific H-bonds are observed (over a total of 5000 frames since each frame is recorded every 4 ps over the last 20 ns of the 600 ns NPT simulations). The H bonds were identified between the side chains of each amino acid and each DNA nucleotide, using a distance cutoff of 3.5 Å and an angle cutoff of 135°. On the other hand, on the residue-specific protein–DNA contact maps (Figure 6a and b, bottom panels), the pixel darkness reflects the shortest average distance (in Å) between atoms of the amino acid and the nucleotide, with darker pixels indicating closer proximity.

**Figure 7:**
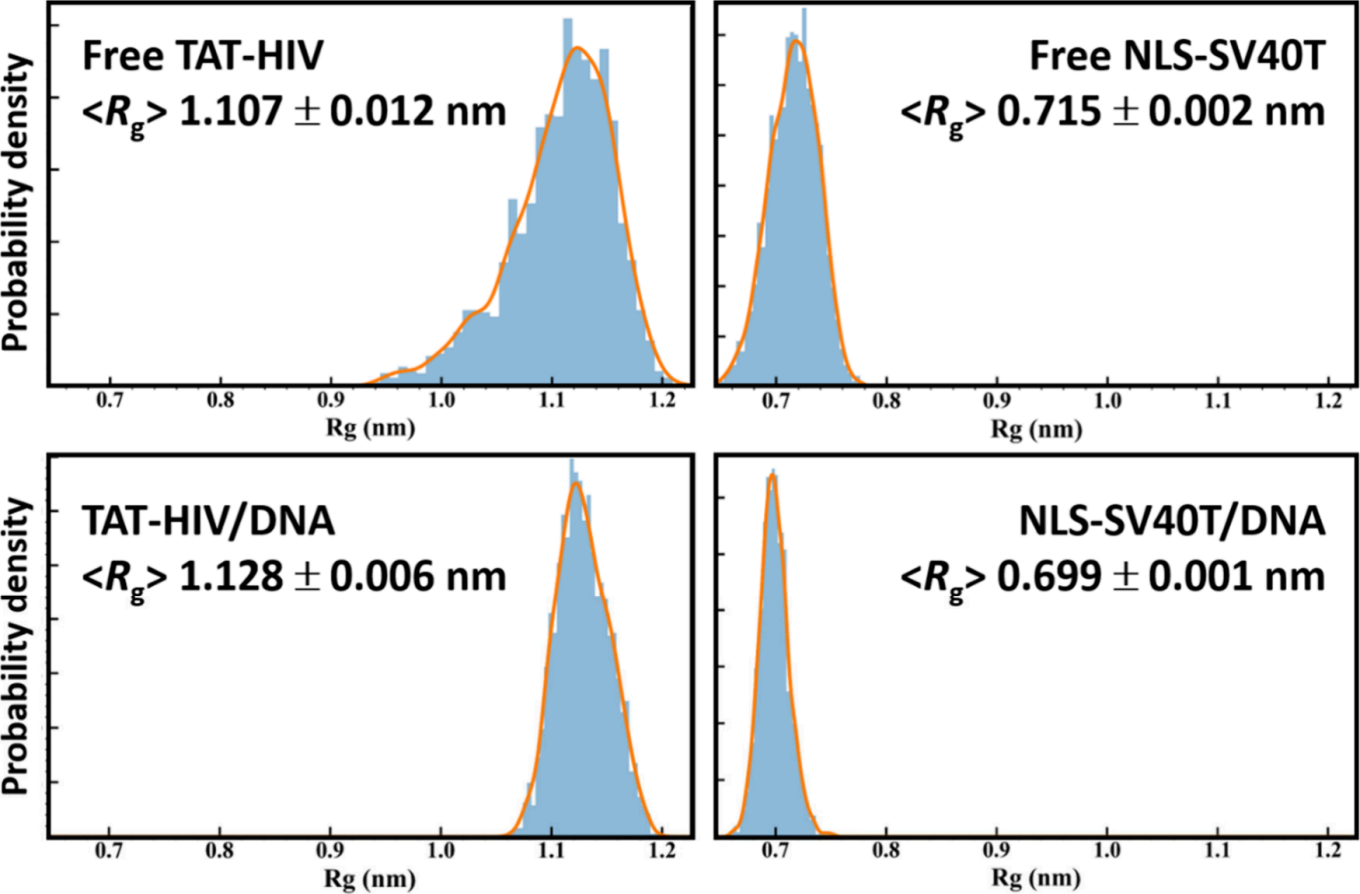
Probability distributions of the radius of gyration (*R*_g_) estimated for free (top) and DNA-bound (bottom) of TAT-HIV (left) and NLS-SV40T (right) over the last 20 ns of the 180 ns (free) or 600 ns (DNA-bound) MD simulations.

These H-bond and contact maps (Figure 6 combined with the residue identification in Figure 4) reveal that, although almost all the amino acids present in the peptides [Arg (R), Lys (K), Gln (Q), and the protonated N-termini Gly (G) and Pro (P)] participate in forming H bonds with DNA, the cationic Arg (R) residues dominate the H-bond interaction. The R92, R95, and R96 residues of TAT-HIV make persistent (∼100%) H bonds with the DNA nucleotides 6 and 7, while the R88 and R91 residues form other persistent H bonds with the DNA nucleotides 32 and 34 (Figure 6a). This again supports the strong bidentate binding of TAT-HIV to DNA (Figure 5a–c), which is consistent with the binding model found in our previous work on a protamine-mimicking arginine-rich cationic peptide of RRRSRRRS to a DNA duplex model of (GC)_20_.^73^ In the case of NLS-SV40T, R91 is the only residue which makes a persistent H bond with the DNA nucleotide 4, while the Lys residues (K88, K89, and K92) form much weaker H bonds with the DNA nucleotides 6 and 36–38 (Figure 6b). This indicates that NLS-SV40T makes weaker bonds to DNA but stays mostly at the major groove of DNA composed of the nucleotides 4–6 and 36–38 (Figure 5b, c). It is interesting to see that the H bonds formed by another cationic residue Lys (K), i.e., K88 and K89 of TAT-HIV as well as K88–K90 and K92 of NLS-SV40T, are not as strong as those formed by Arg (R). Compared to the guanidinium terminal group in the side chain of Arg (R), the ammonium terminal group in the side chain of Lys (K) appears to limit its H bonding with DNA. It does not come close enough to form strong H bonds to the DNA strands, probably because this hydrophilic ammonium terminal group prefers being hydrated in the water phase.

These maps again show more stable binding for TAT-HIV/DNA peptiplex (Figure 6a) than for the NLS-SV40T/DNA peptiplex (Figure 6b). The weaker H bonds of NLS-SV40T with DNA can be attributed to the smaller number of positively charged amino acids, particularly Arg (R), and its smaller size (7-aa) which make it easy to position itself into the major groove of the DNA. On the other hand, the 13-aa TAT-HIV is probably too long and its six planar guanidinium terminal groups of Arg (R) are probably too bulky to fit within a single groove of DNA. However, the length of TAT-HIV (13-aa with six Arg residues) makes it easy to form strong binding with both major and minor grooves of DNA as well as DNA backbones.

#### Peptide Conformation (Radius of Gyration)

In order to evaluate the structural compaction and conformational stability of the peptides upon DNA binding, the radius of gyration (*R*_g_) was calculated for each peptide before and after binding to DNA over the last 20 ns of the 180 ns (before) and 600 ns (after) runs. Their probability distributions are shown in Figure 7. The *R*_g_ provides a quantitative estimate of the overall size and shape of a protein. Lower *R*_g_ values correspond to more compact and globular conformations, whereas higher *R*_g_ values indicate more extended, flexible or dynamically fluctuating structures.

The *R*_g_ analysis reveals distinct differences between the two DNA-peptide complexes. The shorter NLS-SV40T (Figure 7, right), which consistently maintains a lower and narrower *R*_g_ distribution than the longer TAT-HIV (Figure 7, left), shows even lower and narrower distribution (0.715 ± 0.002 nm to 0.699 ± 0.001 nm; Figure 7, left, top to bottom), i.e., becomes more compact, globular, and structurally stable, after binding to DNA. The tight distribution of *R*_g_ values suggests limited structural fluctuations, supporting the tendency of NLS-SV40T to adopt a stable conformation most likely inside the major groove of DNA.

In contrast, the longer TAT-HIV (Figure 7, left), which displays a noticeably higher and broader *R*_g_ distribution than NLS-SV40T, shows even higher but much narrower distribution upon DNA binding (1.107 ± 0.012 nm to 1.128 ± 0.006 nm; Figure 7, right, top to bottom). However, the *R*_g_ distribution of TAT-HIV/DNA is still broader than the distribution of NLS-SV40T/DNA. This indicates that TAT-HIV remains extended and flexible after binding to DNA, which is consistent with its bidentate binding mode with mobile ends, in particular near the protonated N-terminus G87. This feature allows TAT-HIV to explore a wider conformational space, consistent with its known ability to form multiple transient contacts with multiple DNA duplexes. Overall, the *R*_g_ results demonstrate that NLS-SV40T becomes more compact upon DNA binding, whereas TAT-HIV maintains a more dynamic profile which may contribute to the differing binding modes observed between the two peptides.

#### Secondary Structure

The Ramachandran plots of the TAT-HIV and NLS-SV40T peptides, free and DNA-bound, were analyzed over the last 20 ns of the 180 ns (free) or 600 ns (DNA-bound) NPT simulations (Figure S15). In principle, their secondary structures can be identified by these Ramachandran plots.^46^ However, the Ramachandran plots from 12 independent simulations on each peptide show such a large variation, probably due to the intrinsic disorder of these peptides, that it would be risky to draw any conclusion on their secondary structures including any comparison with experimental CD spectra. This high conformation variability aligns with the difficulties found in the analyses of CD data via Dichroweb, and it could be related to the strongly disordered and complex nature of these peptides.

Instead, the DSSP analysis^53^ was applied to assign secondary structures of peptides appearing during the last 20 ns of the long (180 ns for free and 600 ns for DNA-bound) simulations (Table 3). Table 3 (as well as Table S8) reveals that both TAT-HIV and NLS-SV40T, which are typical intrinsically disordered peptides, are still disordered, i.e., in the loop and bend states, even after binding to DNA, in agreement with the CD and SAXS experiments. However, a small amount of secondary structure, polyproline (PP) II helix, evolves upon DNA binding. The change upon DNA binding is more prominent with NLS-SV40T (14.6% to 29.6%) than TAT-HIV (33.3% to 35.6%), in agreement with the experimental finding.

**Table 3:** DSSP Secondary Structure Assignments^a^ ^,^ ^b^

	TAT-HIV		NLS-SV40T	
secondary structure	free (%)	bound (%)	free (%)	bound (%)
loop (no secondary structure)	58.1 ± 0.8	53.6 ± 0.8	72.6 ± 0.3	58.5 ± 0.1
bend (classed as loop)	8.5 ± 0.3	10.0 ± 0.1	12.87 ± 0.04	11.94 ± 0.02
polyproline II helix	33.3 ± 0.5	36.5 ± 0.5	14.6 ± 0.3	29.6 ± 0.1
turn/β-sheet/α-helix	0.0	0.0	0.0	0.0

a Analyzed over the last 20 ns of the 600 ns NPT simulations.

b Similar to the analysis performed over the last 100 ns (see Table S8).

In summary, MD simulations indicate that TAT-HIV is too long and bulky to enter a single major groove of DNA to form compact structures. Therefore, this highly cationic (+9|e|) R-rich peptide TAT-HIV can form bidentate bonds to both major and minor grooves as well as the backbones of DNA through a number of cationic residues. Most likely, the one terminus (near the neutral C-terminus Q99, in particular) strongly bind to a major groove of DNA and stays inert, while the other terminus (near the protonated N-terminus G87) weakly binds to a minor groove or a backbone of DNA, easily detaches itself from it, extends itself toward the water phase, attracting other DNAs but keeping flexible loose structure. In contrast, the smaller but still cationic (+4|e|) NLS-SV40T peptides can enter a major grooves of DNA to form a compact and rigid structure as a building block for DNA self-assembly. Such differences in DNA aggregation induced by different DNA binding modes of CPP (i.e., bidentate and/or dangling TAT-HIV vs embedded NLS-SV40T) will be explored by MD simulations, either atomistic or coarse-grained, on aqueous solutions of multiple DNA and CPPs (TAT-HIV or NLS-SV40T) mixed at various relative ratios and concentrations, as done in our previous simulations regarding protamine-induced DNA condensation.^38,69–72^

### Cell Interaction and Cytotoxicity Assays

After the structural characterization and mechanistic insights provided by MD simulations, the next step was to test the cell uptake of these nanocarriers. Since the noncovalent strategy is usually carried out with smaller sequences of nucleic acids, such as miRNAs and oligoDNAs, fragments with a few hundred base pairs represent a step forward regarding the size of biomolecules loaded into these complexes.^19,20^ For this purpose, the double stranded DNA was separately marked with YOYO-1, a membrane impermeable dye that intercalates into DNA strands and exhibits strong fluorescence upon binding. Figure 8A shows a representative confocal microscopy image of HeLa cells incubated with NLS-SV40T peptiplexes.

**Figure 8:**
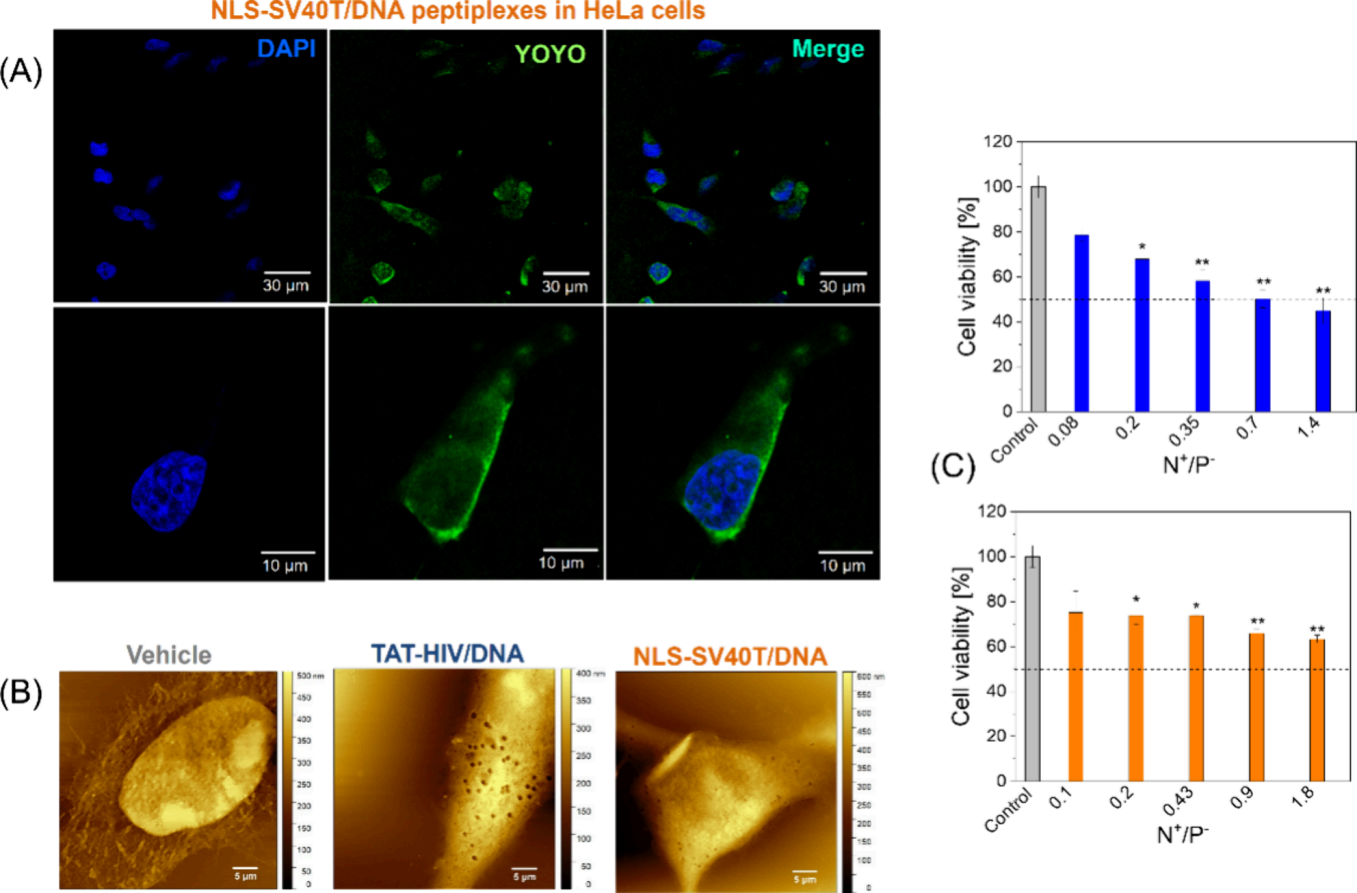
(A) Confocal microscopy images of cells incubated with NLS-SV40T/DNA peptiplexes (5.1 μM peptide and 7.6 μM DNA, N^+^/P^–^ = 2). The blue channel corresponds to DAPI dye staining the nuclei of cells, whereas the green signal is associated with the reported DNA used in the peptiplexes. (B) AFM topography images of HeLa cells incubated with vehicle (PBS buffer) and peptiplexes at extremely high peptide concentrations (100 μg/mL peptide, equivalent to 58.2 μM TAT-HIV + 130 μM DNA or 113.4 μM NLS-SV40T + 170 μM DNA). Membrane disruption is observed for both peptiplexes but is significantly more extensive with TAT-HIV/DNA. (C) MTT data from HeLa cells incubated with NLS-SV40T/DNA and TAT-HIV/DNA peptiplexes. DNA concentration was kept constant at 7.6 μM in all formulations, while the corresponding peptide amount was adjusted to match the different N^+^/P^–^ ratios (*n* = 3; *p < 0.05, **p < 0.01 vs control cells treated with DNA only, determined by Bonferroni-corrected Welch tests).

The image confirms that NLS-SV40T mediates cellular delivery of the fluorescently labeled DNA, although the signal intensity appears relatively low. Confocal scanning imaging along the *z*-direction confirmed the internalization of DNA fragments into cells (Video S4). In contrast, cells treated with TAT-HIV/DNA peptiplexes displayed negligible DNA fluorescence, suggesting that these complexes were unable to translocate nucleic acids across the cell membrane (data not shown). We propose that the TAT-HIV/DNA peptiplexes were unable to deliver nucleic acids into cells because of their self-assembly into long fibrils and large aggregates, whereas the NLS-SV40T/DNA peptiplexes appear organized into more compact assemblies, with sizes in the range of a few tens of nanometers.

We also examined the topography of cell membranes incubated with both peptiplexes at a high peptide concentration. In this case, we prepared complexes at N+/P- = 2 containing peptides at the same mass concentration of 100 μg/mL (equivalent to 58.2 μM TAT-HIV or 113.4 μM NLS-SV40T). The main goal of these assays was to deliberately impose harsh conditions on the cells to stress their membranes and visualize the effect of the different peptiplexes on cell surfaces. In Figure 8B, AFM topography images reveal membrane damage in HeLa cells in the presence of peptiplexes, whereas control cells remain intact. One observes clear membrane disruptions with pits across the cell surfaces, with a much more pronounced effect appearing in the cells incubated with TAT-HIV/DNA peptiplexes compared to NLS-SV40T peptiplexes. Although the AFM images do not provide data from a significant number of cells, and therefore should be considered only qualitative, the membrane disruption is a mechanism widely found in cationic peptides, including antimicrobials, being a phenomenon often associated with cell lysis and possibly explaining the cytotoxicity found at higher peptide concentrations.^74^

In Figure 8C, the viability results from MTT assays show that the cytotoxicity increases with higher N+/P- ratios for both peptiplexes, with a more pronounced effect observed in cells incubated TAT-HIV/DNA. This finding aligns well with the stronger disrupting activity of TAT-HIV indicated by AFM analyses in the membranes of HeLa cells.

## Conclusions

We investigated and compared peptiplexes formed between DNA and two of the most successful CPPs described in the literature, TAT-HIV and NLS-SV40T. These soft biomaterials, composed of peptide-nucleic acids associative complexes formulated through a simple and cost-effective strategy, show strong potential for gene therapy applications due to the increasing recognition of CPPs as safer alternatives to viral vectors. This study represents a rare combination of experimental structural techniques, *in silico* simulations, and *in vitro* cell topography assays to examine and compare nucleic acid vectors based on CPPs. This thorough approach provided insights into the nanoscale order, peptide-DNA binding energetics, secondary structure stability, and impact on membrane morphology.

Our results clearly show that differences in the side chain chemistry of arginine and lysine play a fundamental role in the interaction of CPPs with nucleic acids, affecting the structure of peptiplexes across multiple length scales, with consequences on their bioactivity. At the molecular level, we observed that TAT-HIV displays greater structural flexibility and propensity for acquiring secondary structure upon binding to DNA. This behavior is likely connected to the enhanced capacity of arginine to establish electrostatic and van der Waals interactions, as well bidentate H-bonds, compared to NLS-SV40T/DNA. In addition, we have verified, both experimentally and computationally, that the binding of TAT-HIV to the double helix is much stronger and favorable compared to NLS-SV40T. While the highly positively charged TAT-HIV (+9) is too long and bulky to enter a single major groove of DNA to form compact structures, it can form strong bidentate hydrogen bonds to both major and minor grooves of DNA. In contrast, the smaller but still positively charged NLS-SV40T (+6) can enter the major grooves of DNA to form a compact and rigid structure as a building block for DNA self-assembly. This characteristic potentially enables stronger DNA folding despite the lower binding affinity of NLS-SV40T, assisting the formation of compact, globule-studded assemblies whose inner structure is highly compact with the possible formation of 2D hexagonal DNA phases. Importantly, this combination of tightly packed arrangements, held together by comparatively weaker interactions, may explain the comparatively higher efficiency of NLS-SV40T peptiplexes over TAT-HIV to deliver DNA into cells. Furthermore, the increased charge and H-bonding capacity of TAT-HIV may explain the comparatively higher lytic capacity of its peptiplexes, reinforcing their role as parameters which must be carefully considered in the preparation of peptiplexes. Summarizing, the findings presented throughout this study bring mechanistic insights into the structural landscape of peptiplex materials, improving the rationale that supports the design of peptide-mediated gene delivery systems.

## Supplementary Material











## Notes

Additional experimental and computational details, LC-MS data, electrophoretic runs, concentration measurements, DichroWeb analyses, complementary CD and SAXS data, AFM imaging, supplementary MD snapshots, probability plots, RDF plots, and Ramachandran plots (PDF)
Video S1: TAT-HIV/DNA association (final 20 ns of a 600 ns MD run) (MP4)
Video S2: NLS-SV40T/DNA association (final 20 ns of a600 ns MD run, side view) (MP4)
Video S3: NLS-SV40T/DNA association (final 20 ns of a 600 ns MD runs, top view) (MP4)
Video S4: Confocal Z-scan of a HeLa cell incubated with NLS-SV40T peptiplexes (MP4)
