# Supplementary material for: Structural Dynamics of Peptiplexes Formed between Cationic Cell-Penetrating Peptides and DNA: A Comparative Study on TAT-HIV and NLS-SV40T
Source: ACS Appl Bio Mater. 2026 Jan 20;9(3):1296–310. doi: 10.1021/acsabm.5c01567 (PMC12869476; doi:10.1021/acsabm.5c01567)
Supplement: Supplementary file 1 [file mt5c01567_si_001.pdf]

SUPPORTING INFORMATION

Structural Dynamics of Peptiplexes Formed between Cationic Cell-Penetrating Peptides and DNA: A Comparative Study on TAT-HIV and NLS-SV40T

Lucas R. de Mello†,[1,2] Ibrahim A. Siddiq†,[3] Bianca B. M. Garcia,[1,4] Ian W. Hamley,[2] Karin A. Riske,[1] Sang W. Han,[1] Guillaume Tresset,[5] Yves Lansac,[5,6,*] Yun Hee Jang,[3,*] and Emerson R. da Silva[1,5,*]

[1] Departamento de Biofísica, Universidade Federal de São Paulo, São Paulo 04062-000, Brazil

[2] Department of Chemistry, University of Reading, Reading RG6 6AD, United Kingdom

[3] Department of Energy Science and Engineering, DGIST, Daegu 42988, Korea

[4] Department of Experimental Research, Hospital Israelita Albert Einstein, São Paulo, São Paulo 05653-000, Brazil

[5] Université Paris-Saclay, CNRS, Laboratoire de Physique des Solides, 91405 Orsay, France

[6] GREMAN,UMR 7347, Université de Tours, CNRS, INSA CVL, 37200 Tours, France

Content

Chromatogram and mass spectroscopy data: 2
Electrophoresis: 3
Concentration measurements based on absorptivity at 205 nm: 3
Concentrations in peptiplexes used in MTT assays: 4
SAXS data from peptides and DNA: 5
DichroWeb analyses: 5
CD assays in TFE: 6
CD difference spectra: 6
Gel retardation assays: 7
Supplementary AFM images: 8
Supplementary Molecular Dynamics (MD) Simulation 9

† Equal first authors.

*Corresponding authors: yves.lansac@univ-tours.fr; yhjang@dgist.ac.kr; er.silva@unifesp.br

Chromatogram and mass spectroscopy data:

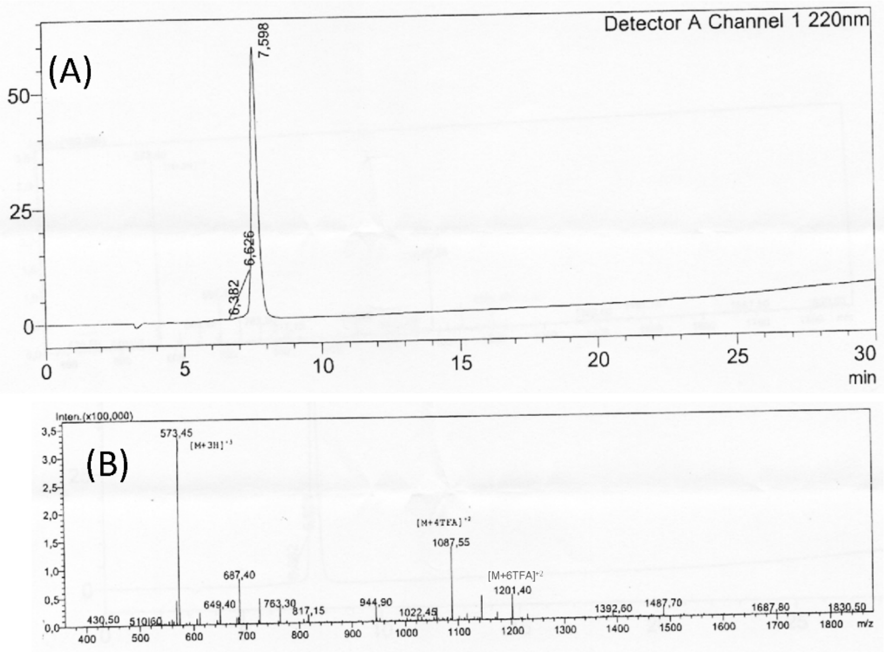

Figure S1. (A) Liquid chromatography and (B) mass spectroscopy data from the TAT-HIV peptide used in this work.

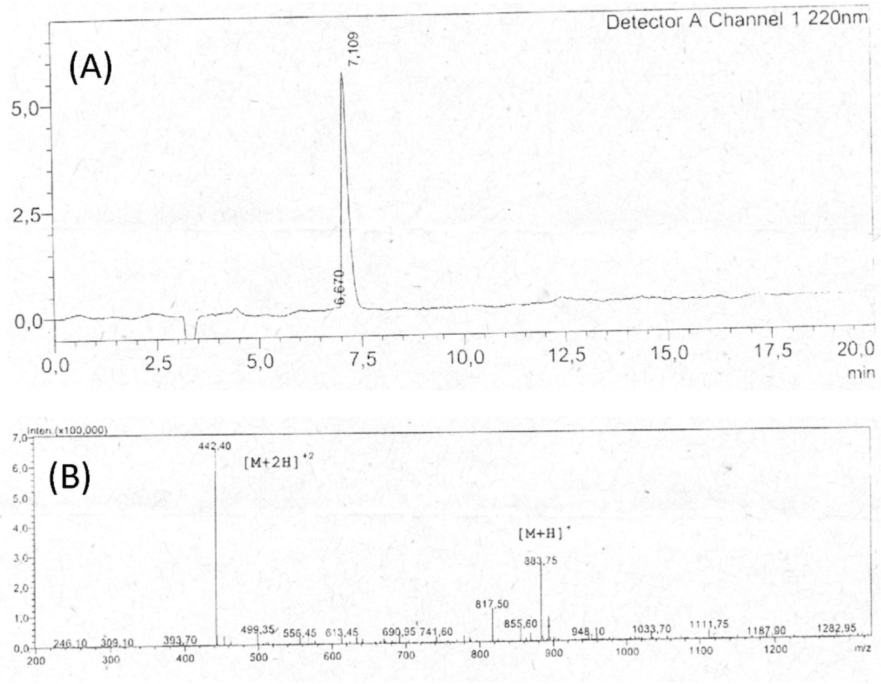

Figure S2. (A) Liquid chromatography and (B) mass spectrometry data from the NLS-SV40T peptide used in this work.

Electrophoresis:

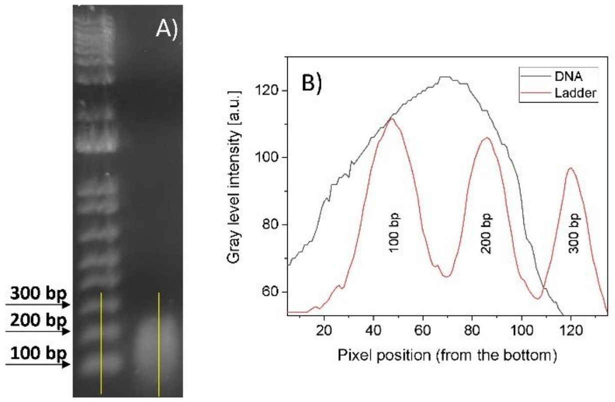

Figure S3. (A) Electrophoretic run of DNA fragments alongside the 100 bp DNA ladder (Thermo Scientific). (B) Gray level profiles along the yellow straight lines shown in (A), indicating that the population of DNA fragments obtained through ultrasonication is mostly composed of strands between 100 and 200 base pairs.

Concentration measurements based on absorptivity at 205 nm:

Considering that the CPPs investigated in this work do not bear tryptophan or tyrosine residues- thus hindering concentration measurements at 280 nm – we followed the procedure proposed by Anthis and Clore [*Protein Sci.* (2013) 22:851-858] to quantify the peptide content in the preparations. The approach is based on UV absorbance measurements performed at 205 nm, a wavelength at which certain amino acid sidechains and peptide bonds exhibit detectable absorptivity. The peptide molar extinction coefficients can be calculated as follows:

$$\varepsilon_{205} = \sum (\varepsilon_i.n_i) + \varepsilon_{bb}.(r-1) \qquad Eq.S1$$

Where ε_i is the molar absorptivity of each amino acid type, ε_{bb} is the molar absorptivity of the backbone peptide bond and r is the number of residues in the strand. Using Eq. S1 alongside the extinction coefficients listed in Table S1, the molar extinction coefficients for TAT-HIV and NLS-SV40T were estimated at **42260 M^{-1}·cm^{-1}** and **18030 M^{-1}·cm^{-1}**, respectively. Finally, the peptide concentrations were determined by using the Lambert-Beer's law: c [M] = $A_{205}/\varepsilon_{205}$. It should be stressed that the method requires careful subtraction of background contributions. In

addition, given the increased dust-induced light scattering at lower wavelengths, we strongly advise filtering buffers with 0.22 µm syringe filters before peptide solubilization and absorbance measurements.

Table S1. Extinction molar coefficients of amino acid sidechains and peptide bonds used to determine molar absorptivity for TAT-HIV and NLS-SV40T.

Sidechain/feature	ε_{205} ($M^{-1}\cdot cm^{-1}$)
Arginine	1350
Glutamine	400
Backbone peptide bond	2780

Concentrations in peptiplexes used in MTT assays:

Table S2. Experimental design of the MTT carried out for TAT-HIV/DNA complexes. The concentration of DNA base pairs was kept at 7.6 µM (or 15.2 phosphate charges):

[TAT-HIV] (µg/mL)	[TAT-HIV] (µM)	N^+ (µM)	N^+/P^-
0.25	0.15	1.3	0.08
0.5	0.3	2.7	0.2
1	0.6	5.4	0.35
2	1.1	9.9	0.7
4	2.3	20.7	1.4

Table S3. Experimental design of the MTT carried out for NLS-SV40T/DNA complexes. The concentration of DNA base pairs was kept at 7.6 µM (or 15.2 phosphate charges):

[NLS-SV40T] (µg/mL)	[NLS-SV40T] (µM)	N^+ (µM)	N^+/P^-
0.25	0.3	1.8	0.1
0.5	0.6	3.6	0.2
1	1.1	6.6	0.43
2	2.3	13.8	0.9
4	4.5	27.0	1.8

SAXS data from peptides and DNA:

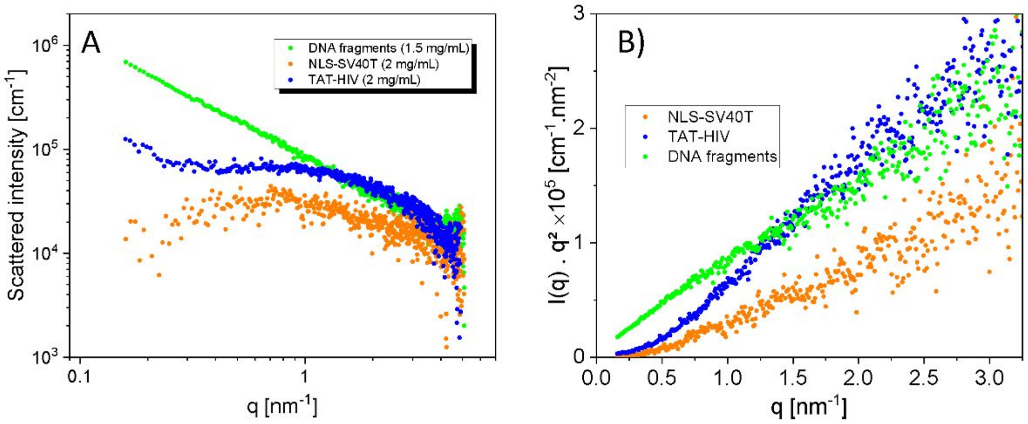

Figure S4. Supplementary SAXS data from samples containing only peptides or DNA fragments. In (A), the data are shown in a log-log representation, while in (B), they are displayed in the Kratky representation. The presence of hyperbolic profiles, with intensities diverging at high q-values in the Kratky plots, is an indication of extended, denatured chains in solution.

DichroWeb analyses:

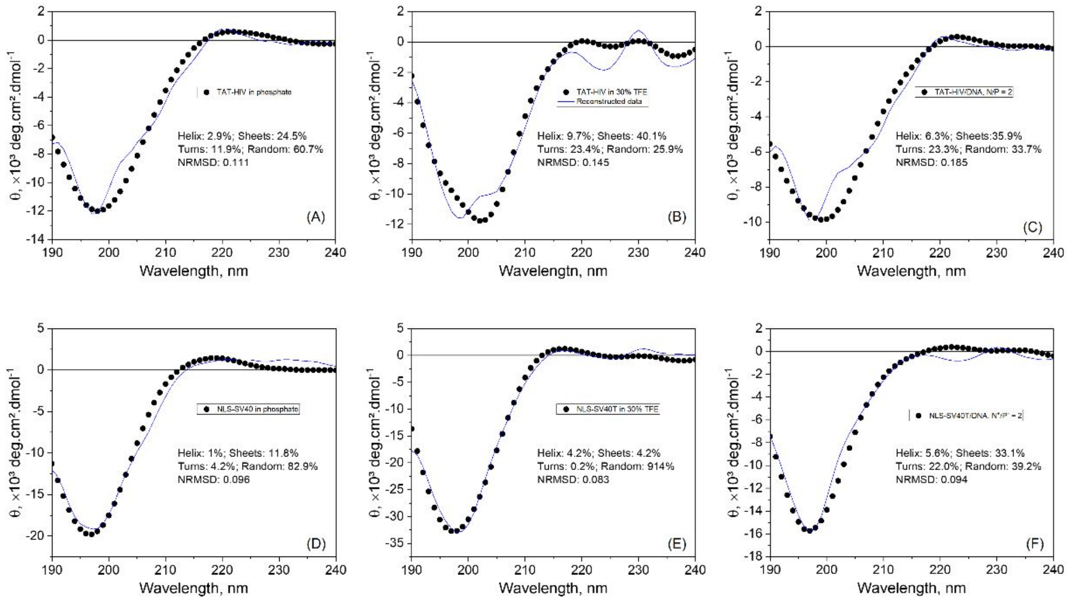

Figure S5. DichroWeb analyses of CD spectra from peptide samples under different conditions. Plots (A)-(C) display spectra of TAT-HIV, while plots (D)-(F) show spectra of NLS-SV40T. The first column corresponds to data collected in aqueous phosphate buffer (A and D, the middle column to data obtained in 30% TFE (B and E), and the right column to the difference spectra of complexes (N/P = 2) after subtraction of the DNA contribution (C and F). The solid blue lines are the reconstructed data according to the best fit using the CONTIN algorithm and the reference dataset 7 (which is optimized for denatured proteins). The percentage values for the different conformations should be regarded as a mere semi-quantitative estimate on secondary structure content. The limitation of the reference dataset to accurately describe spectra from short peptides is evidenced in the case of TAT-HIV samples, likely due to the highest conformational heterogenicity of this peptide.

CD assays in TFE:

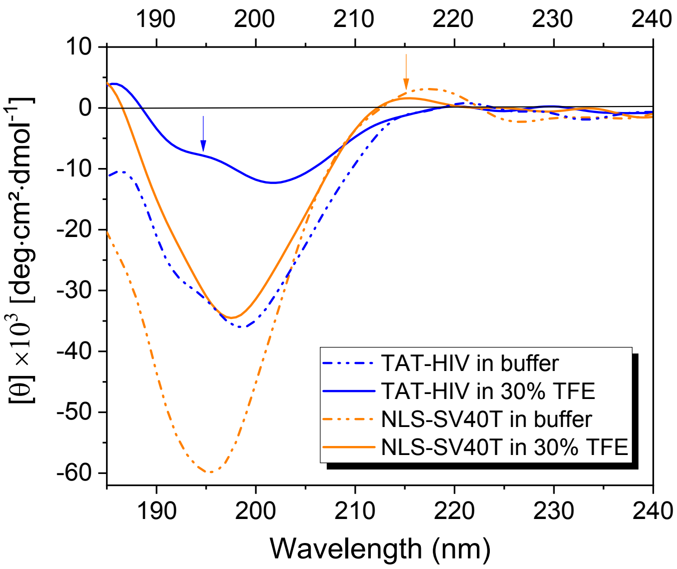

Figure S6. CD spectra from peptide solutions performed in phosphate buffer (30 mM, pH = 7) or in 30% TFE. To avoid buffer absorbance at < 200 nm, it was used a 0.1 mm demountable cuvette with peptide concentrations of 360 µM TAT-HIV and 700 µM NLS-SV40T.

CD difference spectra:

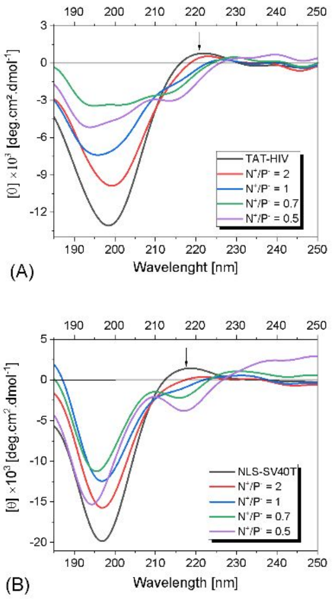

Figure S7. Difference spectra of peptide/DNA complexes prepared at different N^+/P^- ratios. (A) CD data of peptiplexes prepared with TAT-HIV. (B) CD data of peptiplexes prepared with NLS-SV40T. The spectra were obtained by subtracting the contribution of DNA (from a scaled DNA solution spectrum) from the corresponding complex spectrum and normalizing the result by the peptide concentration.

Gel retardation assays:

Experimental procedure: Samples of complexes for gel retardation assays were prepared from a stock of fragmented DNA at 1 mg/mL previously suspended in phosphate buffer (pH 7, 30 mM). For pipetting convenience, this stock was diluted 5-fold, and a volume equivalent to 1 µg of DNA was mixed in Eppendorf tubes together with corresponding amounts of TAT-HIV or NLS-SV40T to achieve charge ratios of N^+/P^- = 0.5, 0.66, 1, and 2. The mixtures were homogenized and left to rest for 30 minutes at room temperature to ensure complex formation. Subsequently, the entire content of the tubes was mixed with loading dye and transferred to the wells of a 2% agarose gel, previously stained with SyBr Green (Thermofisher) for nucleic acid visualization. The first well of each series was loaded with a ladder for DNA size determination, and control wells containing the same amount of DNA without peptide addition was placed on the same gel for comparison. Under these conditions, each well contained a fixed amount of 1 µg of DNA and TAT-HIV amounts of 0.28 µg, 0.38 µg, 0.58 µg, and 1.2 µg to cover the different N^+/P^- charge ratios. In the case of NLS-SV40T, the peptide amounts per well were 0.2 µg, 0.3 µg, 0.45 µg, and 0.9 µg. Electrophoretic runs were performed in a 10 cm apparatus at 100 V for a period of 90 minutes.

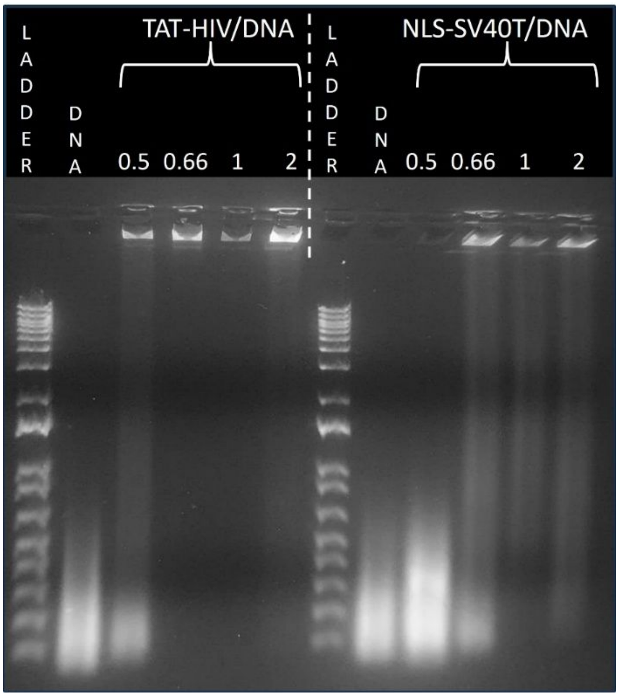

Figure S8. Electrophoretic run assay showing the mobility of complexes prepared with different N^+/P^- ratios. The assay reveals stronger complexation efficiency for TAT-HIV (left) over NLS-SV40T, irrespective of the charge ratio used in the formulation.

Supplementary AFM images:

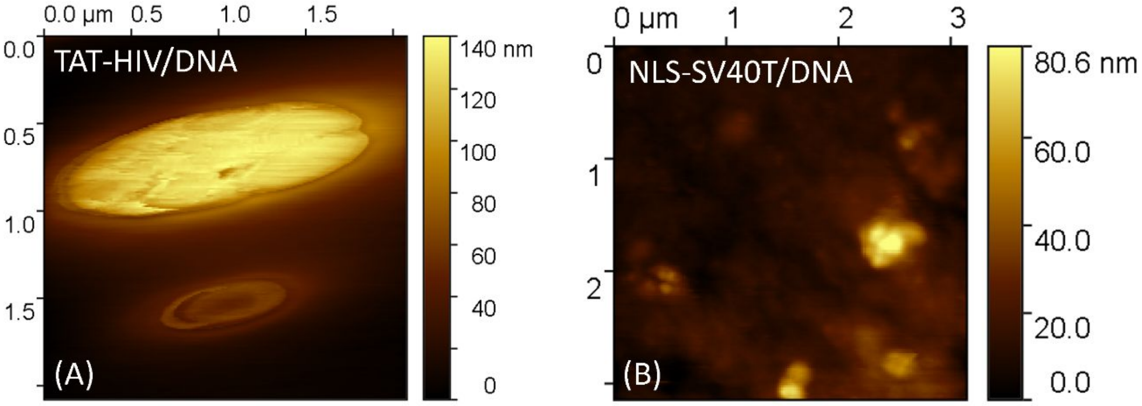

Figure S9. Supplementary AFM topography images of (A) TAT-HIV/DNA and (B) NLS-SV40T/DNA peptiplexes, prepared at a near 2:1 N^+/P^- ratio. The propensity of TAT-HIV/DNA to form elongated clusters markedly contrasts with the tendency of NLS-SV40T/DNA to fold into globular arrangements. Samples were dried on cleaved mica substrates, from solutions containing 1.3 mM peptide.

Supplementary Molecular Dynamics (MD) Simulation

Table S4. Model sequences

Model	Sequence	Charge
B-DNA	5'-d(GCGCGCGCGCGCGCGCGCGCGC)-3'	–44|e|
TAT-HIV	GRKKRRRQRRRPPQ-NH2	+9|e|
NLS-SV40T	PKKKRKV-NH2	+6|e|

Table S5. Initial model systems: free peptide in water

Solute	N(counterions)	N(water)	N(atom)	Box size (nm^3)
TAT-HIV	9 Cl^-	12,419	49,957	9.0 × 9.0 × 9.0
NLS-SV40T	6 Cl^-	11,636	46,697	9.0 × 9.0 × 9.0

In the first set of simulations, two distinct DNA-peptide complexes were prepared for each peptide. Each peptide was initially placed 1 or 2 nm away from the DNA either parallel or perpendicular to the DNA axis. The peptide orientations were allowed to vary with time. After short (a total of 34 ns and 64 ns for NLS-SV40T and TAT-HIV, respectively) NPT simulations, the lower-energy case among the two was selected and submitted to longer (600 ns) NPT simulations (Table S6, Figure S10, and Figure 5).

Table S6. Initial model systems: peptide/DNA in water (Fig. S10)

(1) Solute*	N(counterions)	N(water)	N(atom)	Box size (nm^3)
TAT-HIV/DNA	44 Na^+, 9 Cl^-	15,878	65,211	9.0 × 9.0 × 9.0
NLS-SV40T/DNA	44 Na^+, 6 Cl^-	14,754	60,712	9.0 × 9.0 × 9.0

* Initially each peptide was placed 2 nm away from the DNA.

(2) Solute**	N(counterions)	N(water)	N(atom)	Box size (nm^3)
TAT-HIV/DNA	44 Na^+, 9 Cl^-	15,736	64,643	9.0 × 9.0 × 9.0
NLS-SV40T/DNA	44 Na^+, 6 Cl^-	14,634	60,232	9.0 × 9.0 × 9.0

** Initially each peptide was placed 1 nm away from the DNA.

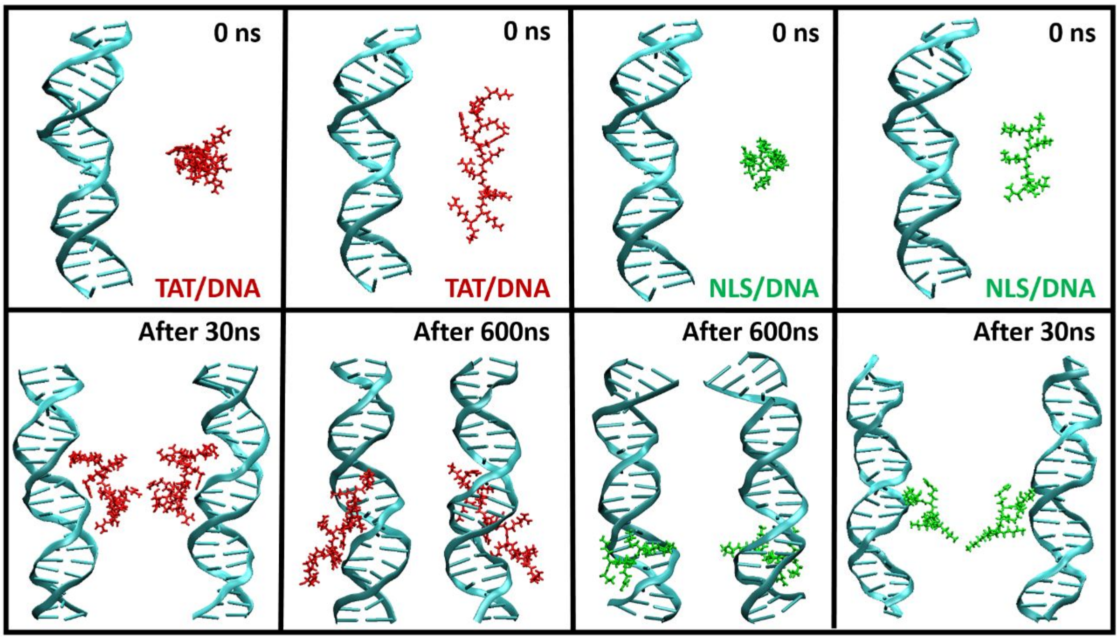

Figure S10. Initial (top) and final (viewed in two directions; bottom) snapshots of unbiased MD simulations on TAT-HIV (red) and NLS-SV40T (green) initially positioned 1~2 nm away from DNA (cyan) in two orientations, parallel or perpendicular to the DNA backbone. Waters, ions, and lattice vectors are hidden for clarity. See Table S6 for system specification.

In the second set of simulations, instead of using the as-built unrealistically-elongated all-trans conformation of TAT-HIV and NLS-SV40T as the initial conformation for DNA binding (as shown in the upper row, Fig. S10), we sample them from a quite long (180-ns) MD simulation on a single free peptide in water. The conformation of the free peptide is evaluated along time by measuring its end-to-end distance (R_{ee}) and radius of gyration (R_g) at each snapshot. From their Gaussian-type distributions or histograms, we select five representative snapshots or conformations, label them 1 to 5 from the most globular one (with the shortest R_g) to the most extended one (with the longest R_g), and use them (T1~T5 for TAT and N1~N5 for NLS) as the 5 different initial conformations in the subsequent DNA-binding MD simulations. The final snapshots shown in Fig. S10, especially those taken after 600-ns runs (columns 2-3), are similar to those shown in Fig. S12 (bidentate binding of TAT-HIV and major groove binding of NLS-SV40T), except only a few cases such as T1m and N2M showing minor-groove binding.

Table S7. Initial model systems: 10 replica for each peptide/DNA (Fig. S11-S12)

Solute*	N(counterions)	N(water)	N(atom)	Box size (nm^3)**
TAT-HIV/DNA	44 Na^+, 9 Cl^-	15,771	65,205	7.7 × 7.7 × 7.7
NLS-SV40T/DNA	44 Na^+, 6 Cl^-	14,728	60,702	7.7 × 7.7 × 7.7

* Each peptide conformer is selected and placed 3 nm away from DNA as shown in Fig. S11.
** Final box size after the unbiased MD simulations described above in Fig. S10 and Table S6

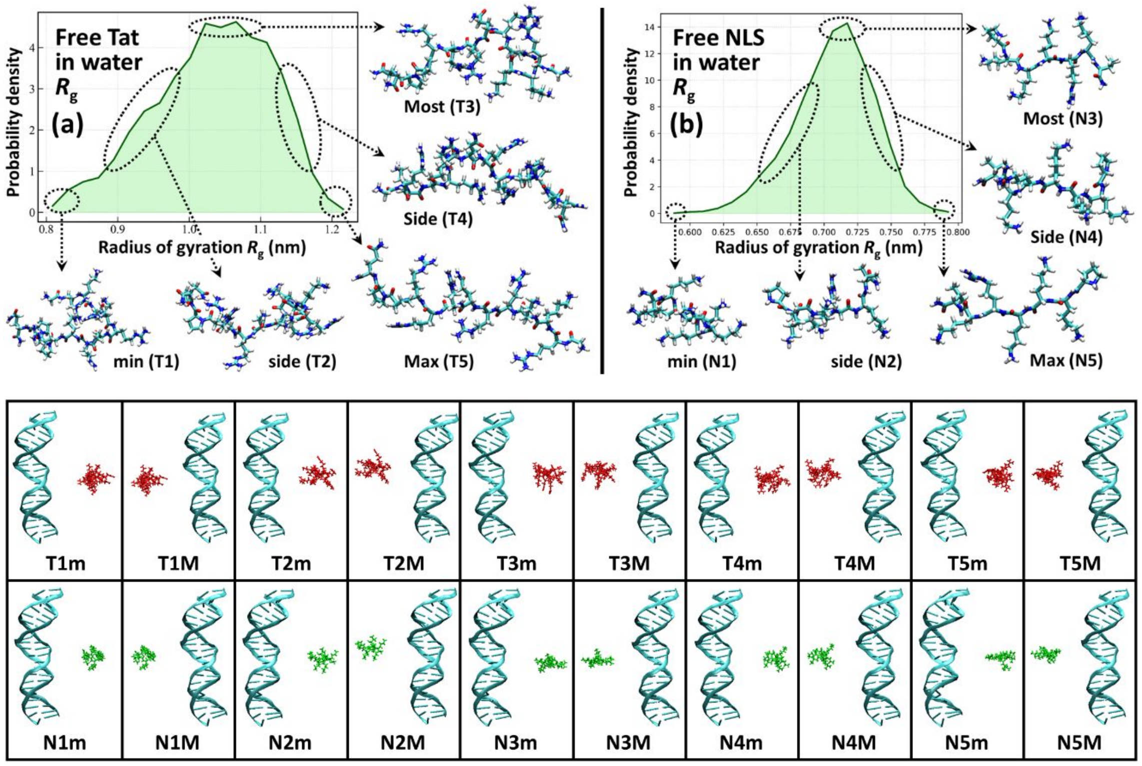

Figure S11. Five conformers of (a) TAT-HIV (T; red) and (b) NLS-SV40T (N; green) selected to represent the whole distribution of free peptide's radius of gyration (R_g), labelled 1 to 5 from the most globular one (with the shortest R_g) to the most extended one (with the longest R_g), positioned 3 nm away from the minor (m) and major (M) grooves of DNA (cyan), and submitted to a series of unbiased MD simulations at 300 K and simulated annealing up to 350 K when necessary. Waters, ions, and lattice vectors are hidden for clarity. The final snapshots are shown below in Fig. S12.

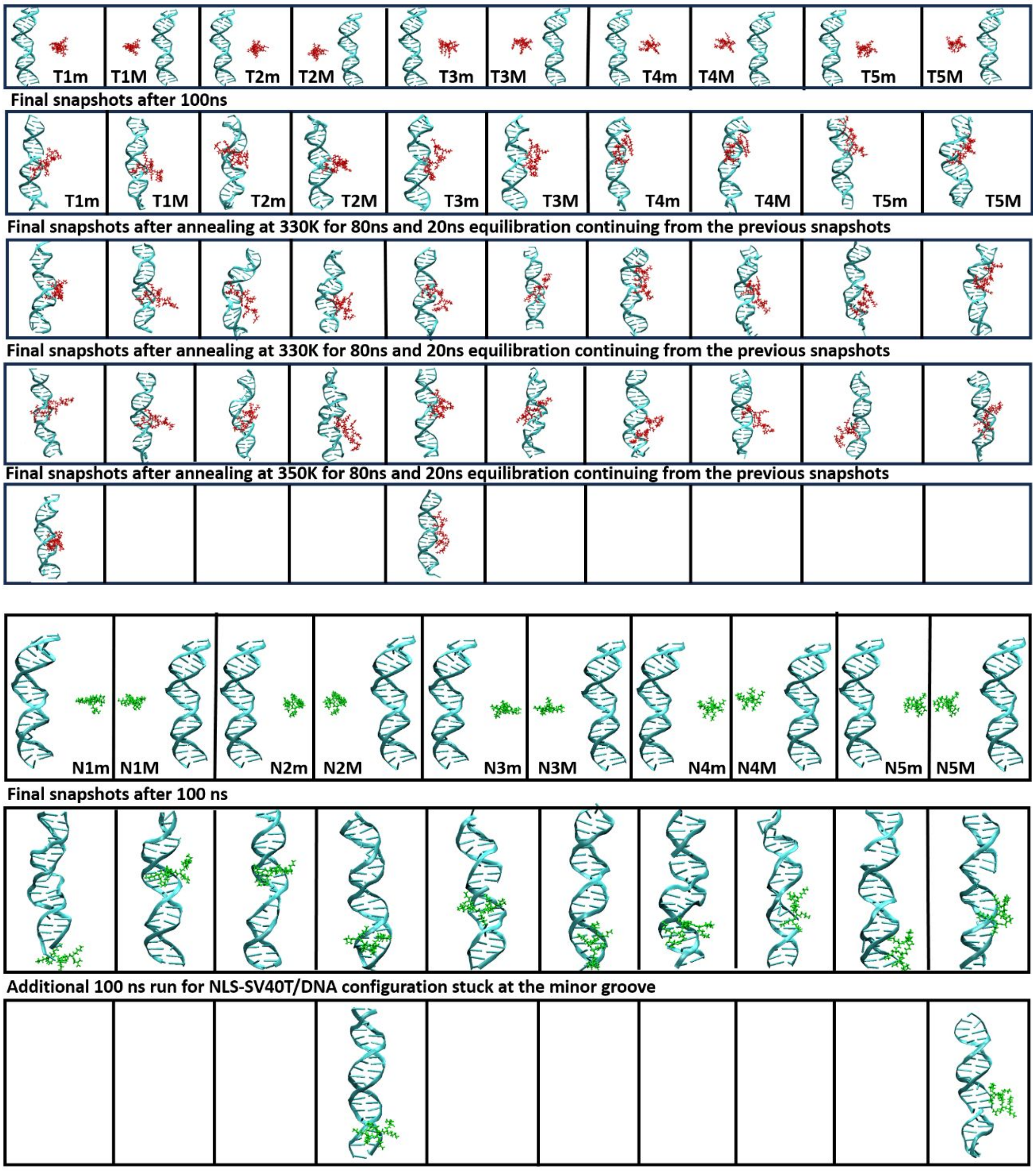

Figure S12. Initial (uppermost row; the same as Fig. S11) and final (lower rows) snapshots of a series of 100-ns unbiased MD simulations at 300 K and subsequent simulated annealing up to 350 K, when necessary, on the 10 initial configurations (or replica) of TAT-HIV (red; T1m to T5M) and NLS-SV40T (green; N1m to N5M) positioned 3 nm away from DNA (cyan). Waters, ions, and lattice vectors are hidden for clarity.

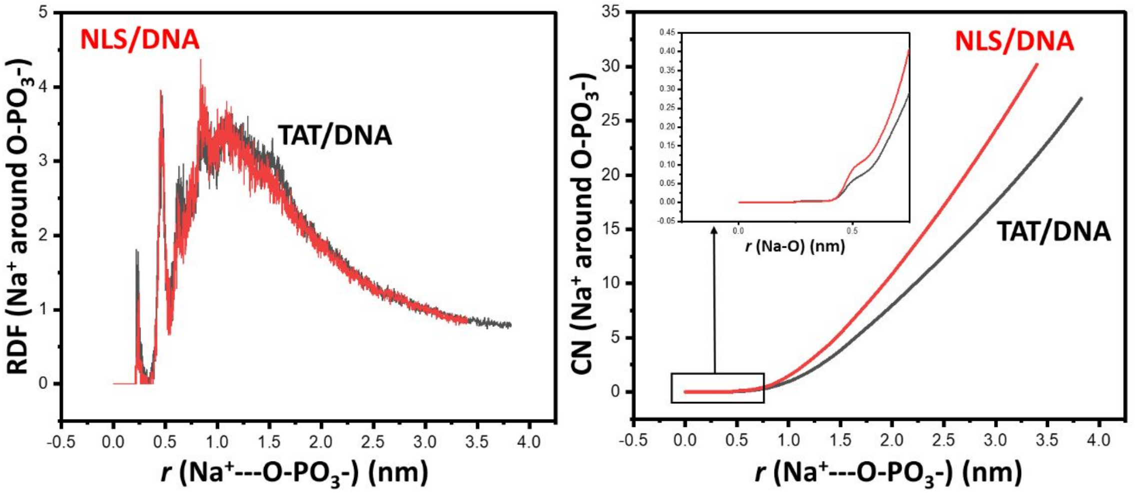

Figure S13. Radial distribution function (RDF; left) and coordination number (CN; right) of Na^+ ions around each phosphate O atom of DNA in the TAT-HIV/DNA (black) and NLS-SV40T/DNA (red) complexes in solution, which were analyzed over the last 20 ns of 600-ns NPT simulations. They are lower in TAT-HIV/DNA than in NLS-SV40T/DNA, indicating a larger amount of Na^+ ion expulsion by TAT-HIV and in turn a higher degree of entropy gain during the TAT-HIV binding to DNA.

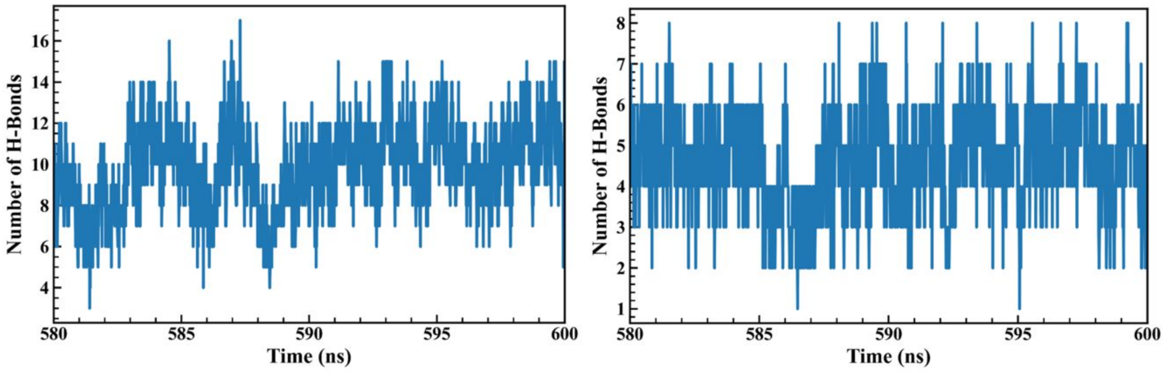

Figure S14. Time evolution of hydrogen (H) bond count for TAT-HIV/DNA (left) and NLS-SV40T/DNA (right) over the last 20 ns of the 600 ns unbiased MD simulations.

Table S8. (for comparison with Table 3 in the main text)

DSSP (last 100 ns)	TAT		NLS	
secondary structure (%)	free	bound	free	bound
loop (no special secondary structure)	59.2 ± 0.2	59.8 ± 0.6	71.12 ± 0.09	56.5 ± 0.1
bend (classed as loop)	15.6 ± 0.3	10.8 ± 0.1	17.0 ± 0.1	12.41 ± 0.02
polyproline II helix	24.7 ± 0.3	29 ± 1	11.7 ± 0.1	31.0 ± 0.1
turn/β-sheet/α-helix	0.0	0.0	0.0	0.0

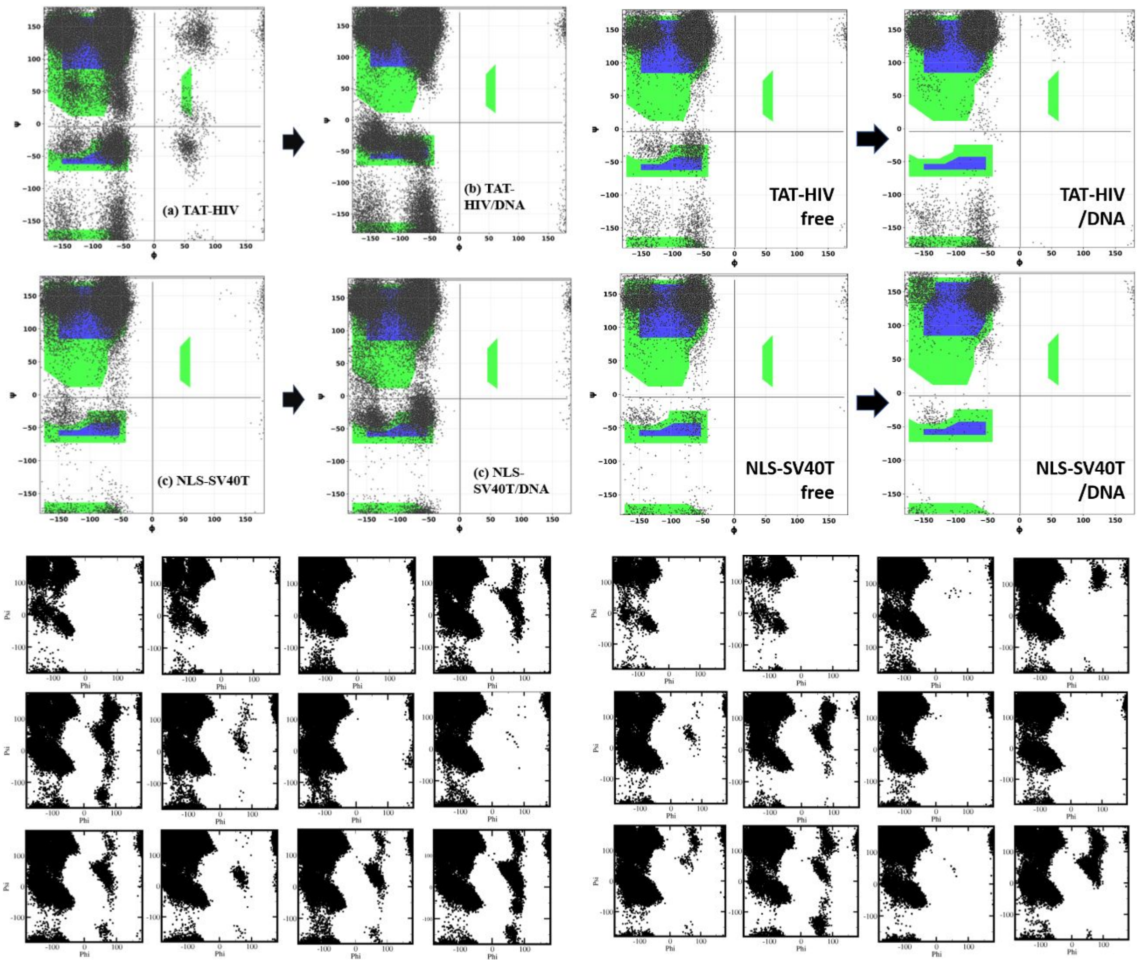

Figure S15. [Top] Ramachandran plots (drawn with AmberTools24) of TAT-HIV and NLS-SV40T before and after binding to DNA in water, analyzed over the last 20 ns of short (left; 20 ns) and long (right; 180-ns for free and 600-ns for DNA-bound) NPT simulations. Typical regions of favorable and highly favorable ϕ and ψ torsion angles are indicated in green and blue, respectively. [Bottom] Ramachandran plots (drawn with GROMACS tools) of DNA-bound TAT-HIV (left) and NLS-SV40T (right), analyzed over the last 20 ns of 12 independent simulations.
